# Unraveling the neuroimmune interface in chronic pain—the association between cytokines in the cerebrospinal fluid and pain in patients with lumbar disk herniation or degenerative disk disease

**DOI:** 10.1097/j.pain.0000000000003175

**Published:** 2024-02-05

**Authors:** Alexander H.C. Rosenström, Aisha Siddiqah Ahmed, Kim Kultima, Eva Freyhult, Svante Berg, Alex Bersellini Farinotti, Vinko Palada, Camilla I. Svensson, Eva Kosek

**Affiliations:** a Department of Surgical Sciences, Uppsala University, Akademiska Sjukhuset, Uppsala, Sweden; b Department of Molecular Medicine and Surgery, Karolinska Institute, Karolinska University Hospital, Stockholm, Sweden; c Department of Medical Sciences, Uppsala University, Akademiska Sjukhuset, Uppsala, Sweden; d Department of Physiology and Pharmacology, Karolinska Institute, Karolinska Institutet, Stockholm, Sweden; e Department of Cell and Molecular Biology, Uppsala University, Uppsala, Sweden; f Department of Clinical Neuroscience, Karolinska Institute, Stockholm, Sweden. Palada is now with the Department of Physiology, University of Helsinki, Helsinki, Finland

**Keywords:** Lumbar disk herniation, Degenerative disk disease, Neuroinflammation, Chronic pain, Neuroimmune interface, Low back pain, Radiculopathy

## Abstract

Recent evidence highlights the importance of the neuroimmune interface, including periphery-to-central nervous system (CNS) neuroimmune crosstalk, in chronic pain. Although neuroinflammatory processes have been implicated in central sensitization for a long time, their potential neuroprotective and analgesic effects remain relatively elusive. We have explored the relationships between cytokine expression and symptom severity, and candidates for periphery-to-CNS crosstalk. Patients with degenerative disk disease (DDD) (nociceptive pain) or patients with lumbar disk herniation (LDH) with radiculopathy (predominantly neuropathic pain) completed questionnaires regarding pain and functional disability, underwent quantitative sensory testing, and provided blood and cerebrospinal fluid (CSF) samples. Proximity extension assay (PEA) was used to measure the levels of 92 inflammatory proteins in the CSF and serum from a total of 160 patients and controls, and CSF/serum albumin quotients was calculated for patients with DDD and patients with LDH. We found signs of neuroimmune activation, in the absence of systemic inflammation. Regarding periphery-to-CNS neuroimmune crosstalk, there were significant associations between several cytokines and albumin quotient, despite the latter being primarily at subclinical levels. The cytokines CCL11, CD5, IL8, and MMP-10 were elevated in the CSF, had positive correlations between CSF and serum levels, and associated in a nonlinear manner with back, but not leg, pain intensity in the LDH, but not the DDD, group. In conclusion, we found evidence for neuroimmune activation in the CNS of both patient groups in the absence of systemic inflammation and signs of a communication between CSF and serum. Complex and disease-specific associations were found between cytokines in CSF and back pain intensity.

## Abstract 2

Supplemental Digital Content is Available in the Text.

A cross-sectional study finding elevated cerebrospinal fluid protein levels in patients with degenerative disk disease and lumbar disk herniation, suggesting neuroimmune activity. The interaction between neuroimmunity and pain was complex.

## 1. Introduction

The so-called “neuroimmune interface” has emerged as a key subject in chronic pain. It is a theory describing the bilateral communication between the nervous and immune systems, both centrally and in the periphery. After noxious stimuli, microglia, the resident immune cells of the central nervous system (CNS), are activated.^32^ They change the neuroenvironment by cytokine release, which leads to several downstream effects. These are, for example, activation of other glial cells such as astrocytes and oligodendrocytes,^32^ and alteration of the permeability of the blood–brain barrier (BBB).^59^ By altering the BBB, cytokines can more readily be transported from the periphery to the CNS and vice versa, enhancing neuroimmune communication.^24,33,59^ Furthermore, glia activation has been reported to induce central sensitization and increased pain sensitivity in animal pain models^67^ and has been documented in patients with chronic pain.^2,45^

Elevated cytokine levels in the cerebrospinal fluid (CSF), often referred to as *neuroinflammation,* have been reported in patients suffering from neuropathic,^7,11,40,52^ nociceptive,^19,37,53^ as well as nociplastic pain.^2,8,37^ These include chemokines involved in neuron-to-glia signaling, such as fractalkine^8,53^ as well as chemokines released by glia and other immunocompetent cells and known to increase the excitability of (peripheral) nociceptive afferents (eg, IL8).^7,37,38^ In addition, in patients with chronic low back pain (LBP)^52^ as well as osteoarthritis (OA),^38,53^ positive associations between CSF and serum concentrations were found for several of these cytokines, indicating their possible role in neuroimmune crosstalk between periphery and the CNS.

Traditionally, nociceptor sensitization has been deemed a result of “proinflammatory” cytokines such as IL1b, IL6, IL8, TNFa, IFNy, and CX3CL1, and neurotrophins such as NGF and BDNF. By contrast, “anti-inflammatory” cytokines such as IL4 and IL10 have been regarded as antinociceptive and/or inhibitory.^9,20,23,33,62^ In contrast to this idea, our research group reported that patients with knee pain because of OA had a *negative* correlation between pain intensity or severity of knee-related symptoms and the CSF levels of several cytokines, including IL6, IL8, CX3CL1, and beta-NGF.^53^ Thus, we could demonstrate that neuroimmune interaction per se is not always associated with increased pain intensity. Instead, our results suggested a context- and possibly time-dependent role for neuroinflammatory processes—a role that could also involve attenuation of pain.

This study aims to further elucidate the relationship between cytokine release and pain by identifying cytokines associated with pain intensity or related symptoms, exploring proteins involved in neuroimmune crosstalk. To disentangle differences between nociceptive and neuropathic pain, 2 well-phenotyped chronic pain cohorts were examined: patients suffering from degenerative disk disease (DDD) and patients with lumbar disk herniations (LDHs) with nerve root pain as their dominant pain component.

## 2. Methods

This study was approved by the ethics committee in Stockholm County, Sweden (2011/2036-31-1). Before inclusion, written informed consent was acquired from all patients. Data from the same cohorts (DDD/LDH patients, healthy controls [HC], and CSF controls) regarding a limited number of cytokines/chemokines (IL6, IL8, and MCP1) assessed with a different method (Meso Scale Discovery immunoassay) have previously been published.^52^

### 2.1. Subjects

#### 2.1.1. Patients

Forty (40) patients with DDD (22 females [F], 18 males [M], average age 44.5 years, range 27-63 years) and 40 patients with LDH (12 F, 28 M, age 41.1 [25–65] years), all waiting for spinal surgery, were recruited from Stockholm Spine Center, Upplands Väsby, Sweden.

##### 2.1.1.1. Patients with degenerative disk disease

Inclusion criteria were age between 25 and 70 years; radiologically confirmed degenerative changes in the lumbar spine with corresponding symptoms, waiting for spinal fusion or disk replacement at 1 to 3 levels; pain duration >1 year; and average weekly pain intensity as rated by visual analogue scale (VAS) > 30 mm on a 100-mm scale.

##### 2.1.1.2. Patients with lumbar disk herniation

Inclusion criteria were age between 25 and 70 years; lumbar disk herniation somewhere between L3 and S1, confirmed with MRT, with symptoms of radiculopathy in agreement with the radiological findings, absence of Modic changes; no more than 50% reduction in any one of the lumbar intervertebral disks, to differentiate patients with LDH from patients with DDD; and pain duration > 1 month with dominating leg pain and average weekly back pain intensity as rated by visual analogue scale (VAS) < 30 mm on a 100-mm scale.

##### 2.1.1.3. Exclusion criteria

Both patient groups had the following exclusion criteria: chronic pain because of any other reason than the above-stated diagnoses (such as fibromyalgia, rheumatic disease, osteoarthritis etc); and previous surgery at investigated levels. In addition, patients with LDH with a previous history of significant back pain were excluded.

#### 2.1.2. Controls

##### 2.1.2.1. Cerebrospinal fluid controls

Patients with noninflammatory neurological symptoms (NINS; n = 40; 23 F, 17 M, age 47.4 [26–73] years) were recruited as CSF controls. No medications were taken on a regular basis and no analgesics had been used on the day of assessment.

They all underwent neurological investigation at Karolinska University Hospital, Stockholm, where they were investigated regarding neurological disease using blood tests (CRP and leukocyte count), CSF analyses (oligoclonal bands, IgG index, and leukocyte count), and MRT of the brain. None of the patients had signs of inflammatory disease in this workup. The most common reason for neurological workup was paresthesia, which was the main complaint in 15 patients. Five CSF controls had pain as the reason for neurological workup, 4 of whom were diagnosed with tension headache and one with migraine. A complete list of CSF-control subjects, including age and reason for neurological workup, can be found in supplemental file A (available as supplemental digital content at http://links.lww.com/PAIN/B991).

##### 2.1.2.2. Serum controls

We recruited 40 HC (24 F, 16 M, age 50.0 [29–65] years) as serum controls by announcing in local newspapers. They were prescreened by telephone and once more on the day of the examination. In addition to the above-stated patient exclusion criteria, healthy controls with other chronic pain conditions or an average weekly pain rating exceeding 20 mm on a 100-mm scale were excluded.

#### 2.1.3. Medication

Among patients with DDD, 7 patients were using codeine, 4 tramadol, 1 buprenorphine, 8 strong opioids, 14 acetaminophen (=paracetamol), 3 antidepressants, and 2 anticonvulsants. Non-steroidal anti-inflammatory drugs (NSAIDs) were used by 5 patients, but because of the planned surgery, this medication had been paused/stopped 2 weeks before. As premedication, patients with DDD were given 1 g of acetaminophen and 20 mg oxycodone orally.

Among patients with LDH, 8 patients were using codeine, 4 tramadol, 5 strong opioids, 17 acetaminophen, 3 antidepressants, and 6 anticonvulsants. NSAIDs were used by 10 patients, but as in the DDD group, this medication had either been paused or stopped 2 weeks before surgery. As premedication, patients with LDH were given 1 g of acetaminophen orally.

In the control groups, no medications were taken on a regular basis and none were given or taken on the day of assessment.

### 2.2. Procedure

During the week before surgery, subjects completed the questionnaires, and underwent pressure algometry and an assessment of conditioned pain modulation (CPM). On the day of surgery, blood and CSF samples were collected before anaesthesia and the surgical procedure. Healthy controls were scheduled for a visit and assessed following the same protocol as the patients, including blood sampling.

#### 2.2.1. Questionnaires

Ratings of global pain (VAS_global), back pain (VAS_back), and leg pain (VAS_leg) were assessed for each patient on the day of inclusion using a 100-mm visual analogue scale (VAS), 0 depicting no pain and 100 the worst imaginable pain.

The Oswestry Disability Index (ODI) was used to determine the subjects' functional disability in relation to their pain. It measures disability regarding 10 topics: pain intensity, personal care, lifting, walking, sitting, standing, sleeping, socializing, travelling, and working (professional work as well as domestic chores). Each topic has a score range of 0 to 5, giving a total maximum of 50 points. The total score is calculated and transformed into “% disability” by multiplying it by 2. The level of disability is thus expressed as a continuous variable between 0% and 100% disability.^18^

#### 2.2.2. Sensory testing protocol

Sensitivity to pressure was assessed using a pressure algometer with a tip of 1 cm^2^ (Somedic Sales AB, Hörby, Sweden). We aimed for a constant pressure increase of approximately 50 kPa/second and asked the subjects to press a button given to them during the testing procedure as soon as the pressure sensation turned to pain. In this study, we assessed pressure pain thresholds (PPTs) at the back (middle between L4-S1). Three PPT assessments were made and the average was calculated as PPT_back and used for further analysis.

#### 2.2.3. Conditioned pain modulation

We used a parallel paradigm CPM with the cold-pressor test as conditioning stimulus (CS) and PPTs as test stimuli (TS). Subjects were instructed to lower their left forearm and hand into an ice water tub (0-1°C) and keep it there as long as possible or for a maximum of 5 minutes. Pressure pain thresholds were assessed at the right *m. quadriceps femoris* before the procedure (PPT_baseline) and every 15 seconds during the procedure. Conditioned pain modulation score was calculated as the relative increase in PPTs at the end of the test (last PPT during CS) compared with the baseline PPT, by dividing the last assessment with the baseline assessment (CPM_score = PPT_end/PPT_baseline).^39^ An increase in PPTs during the CS suggests a normally functioning descending inhibitory system. However, a decrease or absence of increase in PPTs during the CS is indicative of a dysfunctional CPM, which indicates dysfunctional descending pain inhibition.^39,53^

#### 2.2.4. Lumbar puncture and blood samples

Venous blood was collected in 2 × 8.5-mL plastic tubes (BD Vacutainer SST II), incubated for 30 to 40 minutes, and then centrifuged at 2500 rpm for 10 minutes, all in room temperature. Cerebrospinal fluid was collected without additives and immediately centrifuged at 2500 rpm. Both serum and CSF samples were collected, aliquoted, and stored at −80°C, saving both for future analysis.

### 2.3. Proximity extension assay (PEA)

Cerebrospinal fluid and serum samples were analyzed by multiplexed PEA immunoassay using Proseek Multiplex inflammation panel (OLINK Proteomics, Uppsala, Sweden), which allows the simultaneous analysis of 92 inflammation-related proteins across 96 samples.^5,46^ For real-time quantitative polymerase chain reaction (rtPCR/qPCR), a BioMark HD System (Fluidigm, San Francisco, CA) was used.

The 96-well plates were randomized and prepared with 1 μL of either serum or CSF from patients or controls. They were then mixed with 3 μL of solution containing a set of 92 DNA oligonucleotide–conjugated antibodies, one for each of the proteins analyzed. After being incubated at 8°C overnight, 96 μL of extension solution containing PEA enzyme and the reagents for polymerase chain reaction (PCR) were added to the plates and incubated at room temperature for 5 minutes. Finally, rtPCR was performed to amplify and detect the protein levels of the samples according to the instructions in the multiplex panel. The limit of detection (LOD) and the lowest level of quantification (LLOQ) are specified in Supplemental file B (available as supplemental digital content at http://links.lww.com/PAIN/B991).

### 2.4. Enzyme-linked immunosorbent assay

Levels of albumin were measured both in serum and CSF of patients with LDH and patients with DDD using enzyme-linked immunosorbent assay (Invitrogen, Waltham, MA). The protocol was run according to the manufacturer's instructions. Serum samples were diluted 500,000 times, while CSF samples were diluted 1000 to 2000 times. The limit of detection for this assay was 4.92 ng/mL. The obtained albumin concentrations were used to calculate the albumin quotient (Alb_Q_), as a measure of blood–brain barrier permeability, using the following formula:

$${Alb}_{Q}\;\left(mg/g\right)=\frac{\left[CSF\right]\;\left(mg/dL\right)}{\left[serum\right]\;\left(g/dL\right)}$$

Quotients <9 mg/g were deemed normal, while quotients between 9 to 14 mg/g, 14 to 30 mg/g, 30 to 100 mg/g, and > 100 mg/g were considered as slight, moderate, severe, or complete BBB impairment, respectively.^1^

### 2.5. Statistics

The PEA data were expressed on a log2-scale as normalized protein expression (NPX). This allows us to correlate qPCR results to protein concentration, giving us a relative protein quantification between samples. Levels of detection and quantification can be found for each respective protein in supplemental file B (available as supplemental digital content at http://links.lww.com/PAIN/B991). Only proteins that were above the level of detection (LoD) in at least 50% of the samples were included in the analyses. The NPX values were used for further analyses. Potential plate effects were identified using a principal component analysis (PCA). Principal component analysis was performed based on all CSF and serum samples.

Sex differences in protein expression were analyzed using Mann–Whitney *U* test. This is a rank test. Then, to quantify the differences in expression, the difference between median NPX values for male and female subjects were calculated, with positive values indicating higher levels in women.

Associations between protein level and patient group were determined using linear regression. Because of the possible interaction between age, body mass index (BMI), and sex on protein expression, the linear regression was adjusted for these parameters as well as experimental plate, by including them as independent variables in the regression model. Finally, an ANOVA F-test was used to compare group means.

Correlation between CSF and serum samples was assessed using the partial Spearman rank correlation test (implemented in the R-package ppcor^35^), adjusted for plate effect.

Associations between protein levels and clinical parameters (VAS_global, VAS_back, VAS_leg, ODI, PPT_back, and CPM_score) were determined using linear regression. For these tests, pain severity as measured with a 100-mm VAS was discretized into 3 subgroups: 0 to 5 mm = no/mild pain, 6 to 45 mm = moderate pain, and 46 to 100 mm = severe pain. Parameters other than pain, with at least a moderate association with protein level (defined as *P* < 0.05) based on either Spearman rho (age and BMI), Mann–Whitney *U* (sex), or Kruskal–Wallis (plate effect), were adjusted for when determining the association between protein and pain. Finally, associations between clinical parameters and protein level were assessed using ANOVA F-test.

For the analysis of differences in BBB permeability, the albumin quotients were transformed to a log_2_ scale for normalcy, and NPX quotients were calculated for proteins where a significant serum–CSF correlation was found. Because the proteins in the panel are expressed on a log_2_ scale, the CSF/serum quotient is equal to the difference in NPX between the compartments, ie,

$${NPX}_{Q}={NPX}_{CSF}\;–\;{NPX}_{serum}$$

Blood–brain barrier permeability was compared between sexes and between patient groups, using the Mann–Whitney *U* rank test and the Spearman rank correlation test, respectively. The relation between BBB permeability was investigated in relation to quotient of cytokines with a significant serum–CSF correlation in both patient groups, and in relation to their expression levels in the CSF. These analyses were done first in a univariable analysis using the Spearman rank correlation test, and then in a linear regression with simultaneous entering of independent variables, with both log_2_Alb_Q_ and sex as independent variables.

Multiple testing correction was performed using the Benjamini–Hochberg FDR method, correcting for the number of tested proteins, and FDR < 0.1 is considered the significance threshold in the reporting of results.

## 3. Results

### 3.1. Subject characteristics and clinical symptoms

The subject characteristics are presented in Table 1.

**Table 1:** Patient and control characteristics.

	DDD (n = 40) (serum/CSF)	LDH (n = 40) (serum/CSF)	NINS (n = 40) (CSF)	HC (n = 40) (serum)
Females (n)	22 (55%)	12 (30%)	23 (58%)	24 (60%)
Age (years)	44.45 ± 9.91 (27-63)	41.12 ± 10.12 (25-65)	47.35 ± 13.94 (26-73)	50.03 ± 11.35 (29-65)
BMI (kg/m^2^)	25.40 ± 2.29 (19.94-30.12)	26.41 ± 4.33 (16.60-37.22)	N/A	24.23 ± 3.63 (18.3-31.6)
VAS_global (mm)	46 ± 20 (3-79)	47 ± 24 (4-97)	N/A	0.95 ± 2.05 (0-11)
VAS_back	32 ± 20 (4-81)	26 ± 25 (0-95)	N/A	0 ± 0 (0-0)
VAS_leg	6 ± 11 (0-45)	25 ± 27 (0-83)	N/A	0 ± 0 (0-0)
ODI	38 ± 12 (0-68)	39 ± 15 (8-68)	N/A	0.4 ± 0.9 (0-4)
PPT_back	326 ± 169 (55-797)	400 ± 186 (58-908)	N/A	413 ± 169 (129-905)
CPM_score	0.26 ± 0.27 (−0.25 to 0.92)	0.29 ± 0.38 (−0.45 to 1.37)	N/A	0.31 ± 0.40 (−0.49 to 1.57)
AlbQ (mg/g)	3.58 ± 1.02 (1.95-6.38)	4.16 ± 2.46 (1.62-15.39)	N/A	N/A

All values except sex are presented as means ± SD, with minimum and maximum values in parentheses.

Alb_Q_, CSF/serum albumin quotient; BMI, body mass index; CPM, conditioned pain modulation; CSF, cerebrospinal fluid; DDD, patients with degenerative disk disease; HC, healthy controls, for serum analyses; LDH, patients with lumbar disk herniation; NINS, patients with noninflammatory neurological symptoms, which were our control group for CSF analyses; ODI, Oswestry Disability Index, a questionnaire assessing disability on a scale from 0% to 100%; PPT, pressure pain threshold at the back; VAS, visual analogue scale 0 to 100 mm, used for measuring pain in the designated body areas.

Both patient groups reported significantly higher pain ratings than HC in all assessed locations (*P* < 0.0001): global, back, and leg pain and, as expected, patients with LDH had higher ratings of leg pain than patients with DDD (*P* < 0.001).

Compared with HC, both patient groups reported higher levels of disability (ODI) related to back or leg pain (*P* < 0.0001). The levels of disability were not significantly different between patients with DDD and patients with LDH.

Pressure pain thresholds at the painful sites of the back were significantly lower in the DDD group compared with controls (*P* = 0.026). By contrast, PPTs of patients with LDH were not significantly different from those of controls.

There were no statistically significant group differences in age, BMI, CPM score, or albumin quotient.

### 3.2. Cerebrospinal fluid and serum levels of inflammatory proteins

#### 3.2.1. Influence of age and BMI

Two proteins in CSF, LAP-TGFb1 and CXCL9, were significantly associated with increasing age in both patient groups, and age was significantly correlated with higher CSF levels of SIRT2 and Flt3L in the DDD group. Furthermore, significant positive correlations were found between BMI and serum levels of IL17C in the DDD group and between BMI and serum levels of IL6, OSM, IL18R1, HGF, and ENRAGE in the LDH group.

#### 3.2.2. Influence of sex

There were no differences in protein expression in serum nor in CSF between males and females in the DDD group. In the LDH group, there were sex differences in expression of CCL19 in serum (median difference (female − male) = −0.75 NPX, q = 0.018) and in expression of TNFRSF9 in CSF (median difference (female − male) = −0.39 NPX, q = 0.044), both with higher levels in male subjects (Supplementary file E, available as supplemental digital content at http://links.lww.com/PAIN/B991).

Analyzing both patient groups together, male subjects were found to have significantly higher albumin quotients than female subjects (*P* = 0.009).

Based on the findings in sections 3.2.1. and 3.2.2., we concluded that age, BMI, and sex are potential confounders in our study populations. Serum analyses were therefore corrected for age, BMI, sex, and plate effect, while CSF analyses were corrected for age, sex, and plate effect (as we lack BMI data for CSF controls [NINS]), by including them as independent variables in the regression model.

#### 3.2.3. Group differences in cerebrospinal fluid and serum levels of inflammatory proteins

Only proteins with statistically significant group differences in CSF or serum concentrations are presented in this section. A comprehensive overview of all analyzed CSF and serum proteins, respectively, including their full names are listed in the supplemental files B, C, and D (available as supplemental digital content at http://links.lww.com/PAIN/B991). Two samples from the LDH group (one serum, one CSF) and 3 subjects from the NINS group, respectively, and one AlbQ could not be analyzed because of technical errors, which is why the group sizes in Tables 2–5 are different from the ones in Table 1.

**Table 2:** Cerebrospinal fluid proteins with significant group differences between patients and controls, corrected for age, sex, and plate effect.

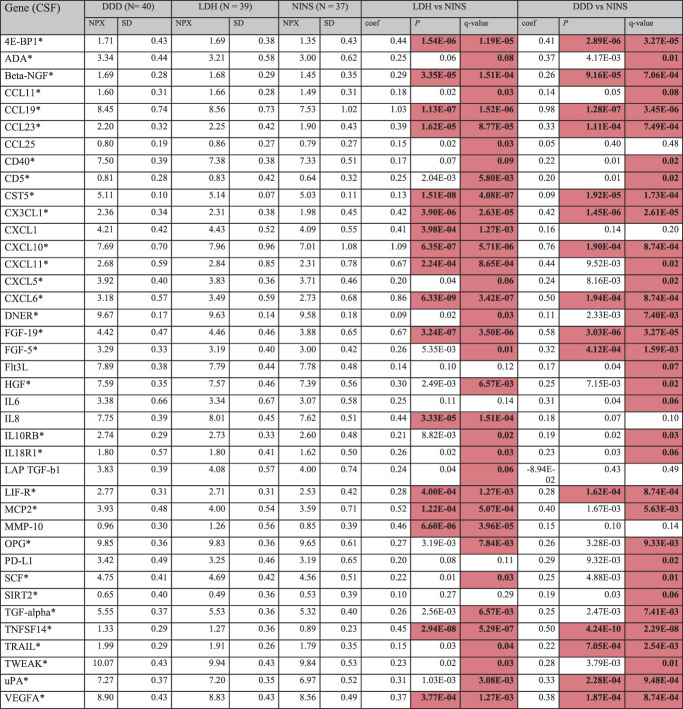

MMP-10 was significantly upregulated in LDH compared with DDD (q = 0.08), but no other proteins were differently expressed between patient groups (not shown). Values in bold indicate a protein is significantly upregulated/downregulated compared with controls, while color indicates direction: red = upregulation compared with the control group, and blue = downregulation compared with the control group. Full protein names and all analyzed proteins can be found in supplemental file B, available as supplemental digital content at http://links.lww.com/PAIN/B991.

*Protein is upregulated compared with controls in both patient groups. NPX values are given in group means.

coef, regression coefficient; DDD, degenerative disk disease; LDH, lumbar disk herniation; NINS, noninflammatory neurological symptoms; NPX, mean normalized protein expression.

**Table 3:** Serum proteins with significant group differences between patients and controls, corrected for age, sex, BMI and plate effect.

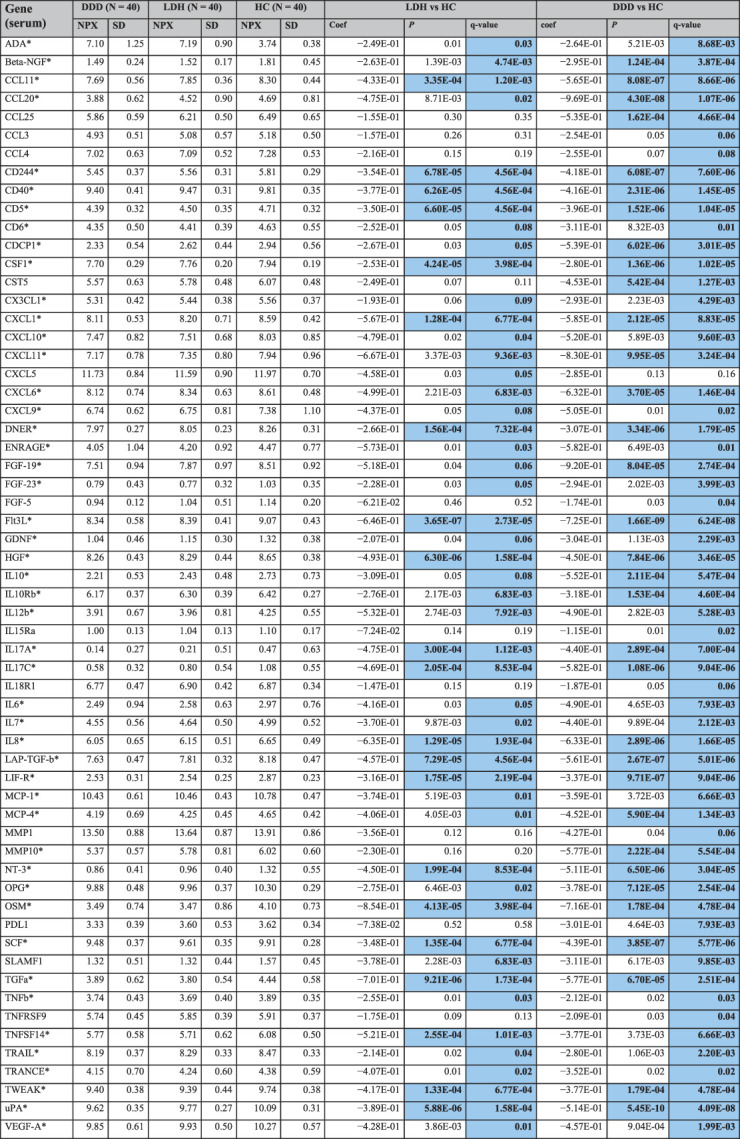

There were no significant differences in expression between DDD and LDH (not shown). Values in bold indicate a protein is significantly up-/downregulated compared with controls, while color indicates direction: red = upregulation compared with the control group, and blue = downregulation compared with the control group. Full protein names and all analyzed proteins can be found in supplemental file B, available as supplemental digital content at http://links.lww.com/PAIN/B991.

*Protein is upregulated compared with controls in both patient groups. NPX values are given in group means.

coef, regression coefficient; DDD, degenerative disk disease; HC, healthy control; LDH, lumbar disk herniation; NPX, mean normalized protein expression.

**Table 4:** Proteins with significant serum–cerebrospinal fluid correlation in expression, analyzed regarding the associations between normalized albumin quotient and expression quotient (cerebrospinal fluid/serum), in patients with degenerative disk disease and patients with lumbar disk herniation.

Protein	NPX quotient ± SD (range; min-max)	Corr coeff	*P*	q-value
Both groups (N = 75)				
CCL11	−6.14 ± 0.37 (1.83; −6.86 to (−5.03))	0.06	0.61	0.75
CCL23	−7.41 ± 0.43 (2.66; −8.80 to (−6.13))	0.27	0.02	0.14
CCL25	−5.22 ± 0.47 (2.28; −6.13 to (−3.85))	−0.07	0.56	0.75
CXCL9	−3.93 ± 0.67 (2.83; −5.08 to (−2.26))	0.20	0.09	0.38
IL12b	−3.04 ± 0.62 (3.16; −4.59 to (−1.44))	0.26	0.03	0.14
IL18	−6.32 ± 0.47 (2.15; −7.43 to (−5.28))	0.34	2.57E-03	0.04
DDD (N = 38)				
CCL4	−3.54 ± 0.52 (2.27; −4.72 to (−2.45))	−0.10	0.54	0.65
CD40	−1.92 ± 0.43 (1.98; −2.97 to (−1.00))	0.17	0.31	0.46
MCP-2	−4.43 ± 0.53 (2.02; −5.51 to (−3.50))	0.02	0.88	0.94
LDH (N = 37)				
CXCL6	−4.88 ± 0.75 (3.27; −6.12 to (−2.85))	−0.05	0.78	0.99
IL8	1.86 ± 0.50 (2.41; 0.38 to 2.78)	0.00	0.99	0.99
IL18R1	−5.13 ± 0.43 (1.78; −6.24 to (−4.46))	0.12	0.46	0.99
MMP-10	−4.48 ± 0.72 (4.11; −6.29 to (−2.18))	0.02	0.92	0.99
CD5	−3.67 ± 0.44 (2.53; −4.69 to (−2.17))	0.04	0.82	0.99
TRAIL	−6.38 ± 0.36 (1.52; −7.10 to (−5.58))	0.07	0.68	0.99
TWEAK	0.54 ± 0.72 (2.85; −0.50 to 2.34)	−0.26	0.12	0.50

Proteins with significant serum–CSF correlation in both groups were analyzed jointly and the remaining proteins were analyzed separately in patients with DDD and patients with LDH. Using the significant proteins listed in Table 6, NPX(Q) was correlated with Alb(Q) using the Spearman rank correlation test.

Alb(Q), albumin quotient; DDD, degenerative disk disease; LDH, lumbar disk herniation; NPX, normalized protein expression.

**Table 5:** Proteins with significant serum–cerebrospinal fluid correlation in expression, analyzed regarding the associations between normalized albumin quotient and cerebrospinal fluid expression of each respective protein, in patients with degenerative disk disease and patients with lumbar disk herniation.

Protein	NPX(CSF) ± SD	Corr coeff	*P*	q-value
BOTH groups (N = 78)				
CCL11	1.63 ± 0.30	0.26	0.02	0.07
CCL23	2.23 ± 0.37	0.39	4.94E-04	3.95E-03
CCL25	0.83 ± 0.23	0.32	3.88E-03	0.02
CXCL9	2.84 ± 0.86	0.24	0.03	0.07
IL12b	0.91 ± 0.37	0.03	0.77	0.85
IL18	1.24 ± 0.40	0.46	2.75E-05	4.39E-04
DDD (N = 40)				
CCL4	3.50 ± 0.52	0.17	0.28	0.35
CD40	7.50 ± 0.39	0.09	0.59	0.59
MCP-2	3.93 ± 0.48	0.14	0.41	0.44
LDH (N = 38)				
CXCL6	3.49 ± 0.59	0.16	0.33	0.67
IL8	8.01 ± 0.45	0.20	0.23	0.61
IL18R1	1.80 ± 0.41	−0.02	0.90	0.90
MMP-10	1.26 ± 0.56	0.07	0.69	0.85
CD5	0.83 ± 0.42	0.06	0.74	0.85
TRAIL	1.91 ± 0.26	0.11	0.50	0.80
TWEAK	9.94 ± 0.43	−0.36	0.03	0.12

The analysis is made jointly for proteins with significant serum–CSF correlations in both groups, otherwise separately in DDD and LDH. Using the significant proteins listed in Table 6, NPX(CSF) was correlated with Alb(Q) using the Spearman rank correlation test.

Alb(Q), albumin quotient; DDD, degenerative disk disease; LDH, lumbar disk herniation; NPX, normalized protein expression.

Compared with the NINS cohort, significant differences (FDR q value < 0.1) in the distribution of NPX mean values for cytokine expression in CSF was found for 34 proteins in the DDD group and 34 proteins in the LDH group, 30 of which overlapped (Table 2). All identified proteins were upregulated compared with the NINS cohort. MMP-10 was significantly upregulated in the CSF of patients with LDH compared with patients with DDD (q = 0.08), but no other proteins were differently expressed between patient groups (supplemental file C, available as supplemental digital content at http://links.lww.com/PAIN/B991).

When compared with HC, significant differences (FDR q value < 0.1) in NPX mean values for cytokine expression in serum were found in a total of 59 proteins in patients with DDD and 49 proteins in patients with LDH, 48 of which overlapped (Table 3). All these were downregulated in serum of patients with DDD and patients with LDH compared with HC. There were no significant differences in protein expression in serum between patients with LDH and patients with DDD (supplemental file D, available as supplemental digital content at http://links.lww.com/PAIN/B991).

### 3.3. Albumin quotients

As a measure of BBB integrity, CSF/serum albumin quotients were calculated for the DDD group (N = 40, mean = 3.59, range 1.95-6.38, SD = 1.02) and the LDH group (N = 39, mean = 4.16, range = 1.62-15.39, SD = 2.46). There was no difference in distribution of Alb_Q_ between the groups (z = −0.061, *P* = 0.951). Using clinical cutoff values, all patients had normal values, ie, Alb_Q_ < 9 mg/g, but one patient in the LDH group had an Alb_Q_ of 15.39, corresponding to a moderate impairment of the BBB. In addition, one patient in the LDH group was excluded from the analyses because of technical issues.

### 3.4. Proteins with significant associations between cerebrospinal fluid and serum

#### 3.4.1. Serum–cerebrospinal fluid correlation

Significant positive associations (FDR q value < 0.1) between serum and CSF levels were found for 6 proteins in both patient groups: CCL11, CCL23, CCL25, CXCL9, IL12b, and IL18. Proteins with significant association specific to the DDD group were CCL4, CD40, and MCP2. Similarly, proteins specific to the LDH group were CXCL6, IL8, IL18R1, MMP-10, CD5, TRAIL, and TWEAK (negative association) (Table 6).

**Table 6:** Proteins with significant serum–cerebrospinal fluid correlation in expression, analyzed with the Spearman rank correlation test, in patients with degenerative disk disease and patients with lumbar disk herniation.

DDD					LDH				
Protein name	Gene	Rho	*P*	q-value	Protein name	Gene	Rho	*P*	q-value
C-C motif chemokine 4*	CCL4	0.48	4.8E-03	0.03					
C-C motif chemokine 11*†	CCL11	0.67	2.0E-05	5.3E-04	C-C motif chemokine 11*†	CCL11	0.62	1.8E-04	2.4E-03
C-C motif chemokine 23†	CCL23	0.68	1.2E-05	5.3E-04	C-C motif chemokine 23†	CCL23	0.55	1.2E-03	0.01
C-C motif chemokine 25*	CCL25	0.53	1.4E-03	0.01	C-C motif chemokine 25†	CCL25	0.72	2.8E-06	5.0E-05
CD-40-L receptor*†	CD40	0.55	9.7E-04	0.01					
					C-X-C motif chemokine 6*†	CXCL6	0.41	0.02	0.09
C-X-C motif chemokine 9*	CXCL9	0.53	1.5E-03	0.01	C-X-C motif chemokine 9*	CXCL9	0.50	3.2E-03	0.02
					Interleukin 8*†	IL8	0.49	4.2E-03	0.03
Interleukin 12 subunit beta*	IL12b	0.49	3.7E-03	0.03	Interleukin 12 subunit beta*	IL12b	0.74	1.1E-06	3.0E-05
Interleukin 18	IL18	0.53	1.5E-03	0.01	Interleukin 18	IL18	0.76	5.0E-07	2.7E-05
					Interleukin 18 receptor 1	IL18R1	0.52	2.2E-03	0.02
					Matrix metalloprotease 10†	MMP-10	0.47	7.2E-03	0.04
Monocyte chemotactic protein 2†	MCP-2	0.43	0.01	0.07					
					T-cell surface glycoprotein CD5 isoform*†	CD5	0.57	6.1E-04	6.6E-03
					TNF-related apoptosis-inducing ligand*†	TRAIL	0.46	7.7E-03	0.04
					Tumor necrosis factor (ligand) superfamily, member 12*†	TWEAK	−0.43	0.01	0.06

* Proteins are significantly downregulated in serum.

† Proteins are significantly elevated in CSF.

#### 3.4.2. Blood–brain barrier permeability and protein expression

To elucidate the effect of changes in BBB permeability on the expression of proteins in the CSF, we correlated log_2_(Alb_Q_) with NPX_Q_ (Table 4) and NPX_CSF_ (Table 5), using the proteins with a significant serum–CSF correlation from section 3.4.1.

Only the IL18 quotient was significantly associated (q = 0.04) with the normalized albumin quotient, when analyzing both patient groups simultaneously. The association was of moderate strength, with a positive correlation coefficient of 0.34. Looking at the NPX quotients in relation to albumin quotients for the proteins that had significant serum–CSF correlation in each respective patient group, no significant associations were found. These findings are summarized in Table 4.

Looking specifically at the expression in CSF, there were significant associations between log_2_(Alb_Q_) and NPX_CSF_ for 5 proteins, namely, CCL11 (q = 0.07), CCL23 (q < 0.01), CCL25 (q = 0.02), CXCL9 (q = 0.07), and IL18 (q < 0.01) (when analyzing both patient groups simultaneously). The correlations were positive with coefficients of 0.26, 0.39, 0.32, 0.24, and 0.46, respectively, suggesting correlations of weak-moderate strength. There were no significant correlations between normalized Alb_Q_ and NPX_CSF_ when analysed in each respective patient group. These findings are outlined in Table 5.

In a linear regression, with log_2_(Alb_Q_) and sex as independent variables, the 6 proteins that showed significant serum–CSF correlation in both patient groups were further analyzed, first regarding the independent variables' effect on NPX_Q_, and then regarding the independent variables' effect on NPX_CSF_.

##### 3.4.2.1. The effect of albumin quotient and sex on NPX_Q_

*R*^2^ values were in the range of 0.01 to 0.19, indicating that only a fraction of the variance in protein quotient is explained by our model. The highest *R*^2^ values were for the regressions on CCL23 and IL18, with *R*^2^ of 0.15 and 0.19, respectively.

The relationship between NPX_Q_ and log_2_Alb_Q_ was always positive, as expected, indicating that an increase in Alb_Q_ is associated with an increase in NPX_Q_. This relationship was significant (*P* < 0.05) between Alb_Q_ and CCL23_Q_ (B = 0.32, *P* = 6.14E-04), IL12B_Q_ (B = 0.36, *P* = 0.009), and IL18_Q_ (B = 0.36, *P* = 3.23E-04).

Regarding the influence of sex on NPX_Q_, there was a significant relationship between sex and IL18_Q_ (B = −0.20, *P* = 0.023), indicating lower BBB permeability in females, which is in line with the univariable analyses done in section 3.2.2.

These results are summarized in Table 7.

**Table 7:** Linear regression with sex and log_2_ albumin quotient as independent variables.

Dependent variable	R square	Independent variable	B (unstandardized)	95% CI	*P*
CCL11 quotient	0.02	Constant Sex log2(AlbQ)	−6.07 −0.08 0.03	−6.52 to (−5.62) −0.26 to 0.10 −0.14 to 0.19	0.355 0.765
CCL11 CSF expression	0.15	Constant Sex log2(AlbQ)	1.59 −0.15 0.13	1.25 to 1.93 −0.28 to (−0.01) 0.01 to 0.25	0.031 0.038
CCL23 quotient	0.15	Constant Sex log2(AlbQ)	−8.11 0.07 0.32	−8.60 to (−7.62) −0.12 to 0.27 0.14 to 0.50	0.459 6.14E-04
CCL23 CSF expression	0.19	Constant Sex log2(AlbQ)	1.73 −0.04 0.29	1.32 to 2.15 −0.21 to 0.12 0.14 to 0.44	0.609 2.30E-04
CCL25 quotient	0.01	Constant Sex log2(AlbQ)	−5.33 0.08 −0.001	−5.66 to (−4.99) −0.15 to 0.31 −0.21 to 0.21	0.51 0.996
CCL25 CSF expression	0.13	Constant Sex log2(AlbQ)	0.62 −0.04 0.14	0.35 to 0.89 −0.14 to 0.07 0.04 to 0.24	0.496 0.006
CXCL9 quotient	0.05	Constant Sex log2(AlbQ)	−4.44 −0.01 0.28	−5.24 to (−3.63) −0.32 to 0.31 −0.02 to 0.57	0.973 0.064
CXCL9 CSF expression	0.07	Constant Sex log2(AlbQ)	2.51 −0.21 0.33	1.53 to 3.48 −0.60 to 0.18 −0.03 to 0.69	0.29 0.069
IL12B quotient	0.10	Constant Sex log2(AlbQ)	−4.02 0.23 0.36	−4.75 to (−2.78) −0.06 to 0.51 0.09 to 0.62	0.125 0.009
IL12B CSF expression	0.01	Constant Sex log2(AlbQ)	0.85 −0.03 0.06	0.71 to 1.23 −0.21 to 0.15 −0.11 to 0.22	0.752 0.49
IL18 quotient	0.19	Constant Sex log2(AlbQ)	−7.35 0.25 0.36	−7.88 to (−6.83) 0.04 to 0.46 0.17 to 0.55	0.019 3.23E-04
IL18 CSF expression	0.22	Constant Sex log2(AlbQ)	1.62 −0.20 0.25	1.36 to 1.88 −0.36 to (−0.03) 0.10 to 0.41	0.023 0.002

Regression models were made for all 6 proteins that exhibit a significant serum–CSF correlation in both patient groups, namely, CCL11, CCL23, CCL25, CXCL9, IL12B, and IL18. Two regressions were done per protein: one with NPX_Q_ as the dependent variable, and one with CSF expression of the given protein as the dependent variable.

##### 3.4.2.2. The effect of albumin quotient and sex on NPX_CSF_

*R*^2^ values were in the range of 0.01 to 0.22, with regressions for CCL23 and IL18 exhibiting the highest *R*^2^ values of 0.19 and 0.22, respectively.

The relationship between NPX_CSF_ and log_2_Alb_Q_ was positive in all analyses, with significant correlations between log_2_Alb_Q_ and CCL11 (*P* = 0.038), CCL23 (*P* = 2.30E-04), CCL25 (*P* = 0.006), IL12B (*P* = 0.009), and IL18 (*P* = 0.002).

When also taking into account Alb_Q_, sex was negatively correlated with CSF expression of CCL11 (*P* = 0.031) and IL18 (*P* = 0.023), indicating higher levels in male subjects.

These results are summarized in Table 7.

### 3.5. Proteins associated with pain and other clinical parameters

**Figure 1.**
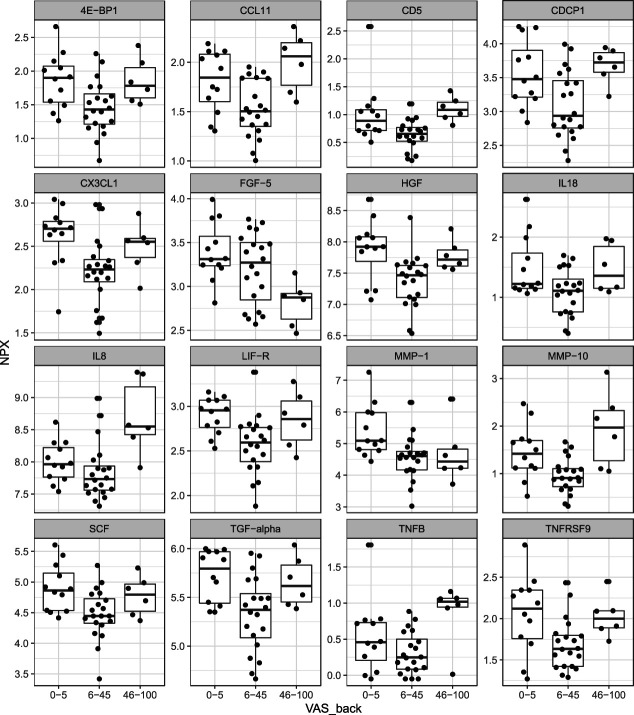
Correlation between pain intensity and expression of CSF proteins. Protein expression was measured in the CSF and correlated with back pain in patients with LDH. Pain was discretized into no/mild pain (VAS 0-5 mm), moderate pain (VAS 6-45 mm), or severe pain (VAS > 45 mm). Please note that patients with LDH were recruited based on having predominant leg pain (radiculopathy), not back pain, hence the limited number of patients with severe pain (VAS > 45 mm). CSF, cerebrospinal fluid; LDH, lumbar disk herniation; NPX, normalized protein expression; VAS, visual analogue scale; VAS_back, rated back pain intensity on a visual analogue scale.

All statistically significant associations between protein levels and clinical parameters are presented in Table 8. Regarding CSF, significant associations were found between 16 proteins and the rated intensity of back pain in the LDH group (VAS_back) (Fig. 1). There were no statistically significant associations between proteins in CSF and symptoms in the DDD group.

**Table 8:** Significant associations between protein expression and clinical parameters.

Protein name	Gene	Type	Group	Variable	F-value	*P*	q-value
CUB domain–containing protein 1	CDCP1	CSF	LDH	VAS_back	5.0	0.013	0.081
Eotaxin-1*†	CCL11	CSF	LDH	VAS_back	4.3	0.023	0.084
Eukaryotic translation initiation factor 4E binding protein 1*	4E-BP1	CSF	LDH	VAS_back	7.1	0.003	0.081
Fibroblast growth factor 5*	FGF-5	CSF	LDH	VAS_back	5.2	0.011	0.081
Fractalkine*	CX3CL1	CSF	LDH	VAS_back	5.2	0.011	0.081
Hepatocyte growth factor*	HGF	CSF	LDH	VAS_back	4.1	0.027	0.091
Interleukin 8*†	IL8	CSF	LDH	VAS_back	7.3	0.002	0.081
Interleukin 18†	IL18	CSF	LDH	VAS_back	4.3	0.022	0.084
Latency-associated peptide transforming growth factor beta-1*	LAP-TGFb	Serum	LDH	VAS_global	12.7	0.001	0.086
Leukemia inhibitory factor receptor*	LIF-R	CSF	LDH	VAS_back	4.9	0.014	0.081
Matrix metalloprotease 1	MMP-1	CSF	LDH	VAS_back	4.6	0.016	0.081
Matrix metalloprotease 10*†	MMP-10	CSF	LDH	VAS_back	4.8	0.015	0.081
Monocyte chemotactic protein 3	MCP3	Serum	DDD	ODI	18.8	1.24E-04	0.009
Stem-cell factor*	SCF	CSF	LDH	VAS_back	4.2	0.023	0.084
T-cell surface glycoprotein CD5 isoform*†	CD5	CSF	LDH	VAS_back	5.8	0.007	0.081
Transforming growth factor alpha*	TGFa	CSF	LDH	VAS_back	5.9	0.006	0.081
Tumor necrosis factor beta*	TNFb	Serum	DDD	PPT_back	12.4	0.001	0.098
Tumor necrosis factor beta	TNFb	CSF	LDH	VAS_back	5.1	0.011	0.081
Tumor necrosis factor receptor superfamily member 9	TNFRSF9	CSF	LDH	VAS_back	4.4	0.02	0.084

* If the expression of the designated protein is significantly different to controls.

† If levels of expression for the designated protein are correlated between serum and CSF.

CSF, cerebrospinal fluid; DDD, degenerative disk disease; LDH, lumbar disk herniation; ODI, Oswestry Disability Index; PPT_spine, pressure pain threshold at the back; VAS_back, rated back pain intensity on a visual analogue scale; VAS_global, rated global pain intensity on a visual analogue scale.

In serum, statistically significant associations were found between general pain (VAS_global) and LAP-TGFb (q = 0.095) in the LDH group, between disability level (ODI) and MCP3 (q = 0.009) in the DDD group, and between pain threshold on the back (PPT_back) and TNFb (q = 0.098) in the DDD group.

No other significant associations were found between pain ratings (VAS_global, VAS_back, and VAS_leg), rated disability (ODI), pressure pain sensitivity (PPT_back), endogenous descending pain inhibition (CPM_score), and protein levels in CSF and serum.

### 3.6. Summary of results

To summarize our results, protein levels in serum and CSF of patients with DDD and patients with LDH were analyzed regarding (1) differences compared with controls, (2) correlations between CSF and serum, (3) associations between albumin quotient and NPX quotient as well as CSF expression, and (4) associations to clinical parameters.

Albumin quotient as a proxy for BBB permeability, while generally being in the “normal” range of clinical cutoff, was significantly higher in males than in females, and significantly correlated with quotients of CCL23, IL12B, and IL18, and with CSF expression of CCL11, CCL23, CCL25, and IL18 (Table 7).

We found a total of 19 significant associations, regarding 18 proteins, between protein expression and symptom severity (Table 8). Of these, 16 proteins are upregulated in CSF and correlate with back pain, while 3 proteins are downregulated in serum and correlate with either general pain (N = 1), disability (N = 1), or pain threshold (N = 1).

**Figure 2.**
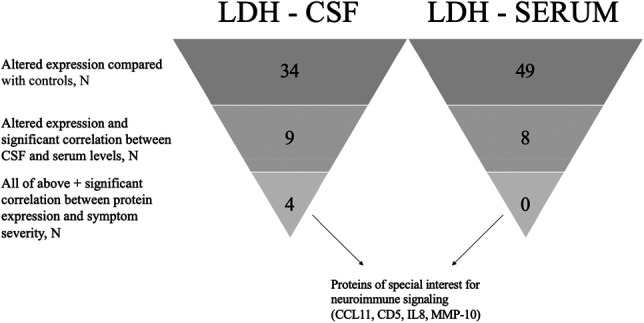
Proteins of interest in neuroimmune signalling. A summary of the results in the LDH group. The proteins in question, namely CCL11, CD5, IL8, and MMP-10, were all upregulated compared with controls and had a significant CSF/serum correlation in expression. CSF, cerebrospinal fluid; LDH, lumbar disk herniation.

Four proteins, all in the CSF of patients with LDH, are upregulated compared with CSF controls, exhibit significant serum–CSF correlation in expression, and are associated with pain intensity (Fig. 1). Therefore, we consider these proteins, namely CCL11, CD5, IL8, and MMP-10, of particular interest regarding neuroimmune interaction (Fig. 2).

## 4. Discussion

### 4.1. Main results

Thirty cytokines were upregulated in the CSF of patients compared with CSF controls (Table 2), indicating neuroimmune activation. Meanwhile, 48 proteins were significantly downregulated in serum of patients compared with healthy controls (Table 3). Regarding neuroimmune crosstalk, 16 cytokines had significant CSF–serum correlation (Table 6), and some correlated with subclinical changes in BBB permeability (Table 7). CCL11, CD5, IL8, and MMP-10 were associated with back pain, but not leg pain intensity in the LDH group (Fig. 1, Table 8). Lumbar disk herniation patients with moderate back pain had lower levels of cytokines compared with no/mild pain (Fig. 1).

### 4.2. Evidence of CNS neuroimmune activation in patients with degenerative disk disease and patients with lumbar disk herniations

In both groups, 30 cytokines were upregulated in the CSF compared with CSF controls, implying neuroimmune activation. Some have been implicated in glial cell activation (eg, fractalkine/CX3CL1) and in modulation of immune cells (eg, CCL23 and CD5). Palada et al.^53^ and Brisby et al.^11^ reported elevated IL8 in the CSF of patients with LDH, which we could replicate, while we could not replicate findings of elevated CSF IL1b, IL10, and TNF in thoracic disk herniation.^4^ Krock et al.^42^ deemed CSF IL8 as important in DDD, while fractalkine was undetectable.

Forty-eight proteins were downregulated in the serum of both patient groups compared with HC. Previous studies have reported upregulation of cytokines such as IL1b,^36^ TNF,^36,41,57,64^ IL2,^36,57^ IL6,^41,57,64,68^ and IL8^57,64^ in the serum of patients with LDH. Time-dependent upregulation of CCL11, CCL3, CXCL1, and CXCL10 has been suggested, as well as an inverse relationship between serum levels of CCL11 and VAS in patients with chronic radicular back pain.^31^ Regarding DDD, CCL5 and CXCL6 have been found to be upregulated in serum,^25^ and serum IL18 has been correlated with DDD severity.^73^ We were not able to replicate these findings. Instead, we found a downregulation of cytokines in the serum of patients and upregulation of cytokines in the CSF—patterns previously reported in OA patients.^38,53^ This can be explained by, eg, time- and severity-dependent alterations of serum cytokines after disk herniation,^11,31,49^ different criteria for the control groups, or differences in pain medication.

Our results provide evidence for increased central and diminished peripheral inflammatory activity that is essentially similar in both patient groups and that tallies with our previous findings.^38,53^ Possible mechanisms include production of cytokines by activated glia,^32^ or by peripheral immune cells, either directly in the CSF after migration, or indirectly by active transportation of cytokines across the BBB, subsequently affecting glial cells and neurons as well as the BBB.^59,66,72^

### 4.3. Neuroimmune interaction: crosstalk between periphery and CNS

Cytokines with positive serum–CSF correlations are interesting because of their possible role in the neuroimmune interface. We found 6 proteins with significant serum–CSF correlations in both patient groups, 4 of which were previously reported in OA patients,^53^ namely, CCL11, CCL25, CXCL9, and IL12b. The consistent findings across 3 different patient groups indicate a potential role of these proteins in periphery-to-CNS crosstalk.

In the CNS, CCL11 is released by activated astrocytes and implicated in microglial migration and oxidative burst.^54,58^ It is induced by IL10 and has been linked to several CNS disorders.^30^ Its main receptor being CCR3, CCL11 also binds to CCR2 as an antagonist in low concentrations and as a partial agonist in higher concentrations.^47^ CCR2 has been linked to increased BBB permeability.^72^ CCL25 has been implicated in inflammatory diseases in the periphery through proinflammatory mechanisms^14,28,61,69,71,74^ but also exhibits protective and homeostatic functions.^70^ Its roles in the CNS and pain remain elusive. IFNγ induces CXCL9, which drives cytotoxic processes and “M1” differentiation through its receptor CXCR3.^13,16,26,50,51,61^ CXCL9 is downregulated in the serum of both our patient groups. Its role in the CNS has not been well established. IL12b induces IFNγ, driving a proinflammatory response,^55,63,65^ and is produced and released by activated microglia after IFNγ stimulation. It is inhibited by various soluble factors released by astrocytes, including IL10.^3^

In summary, we found evidence of downregulation of cytokines related to inflammatory disease (CCL25, IL12, and CXCL9) in the systemic circulation, possibly through an IFNγ-inhibitory effect seeing as CCL25, IL12, and CXCL9 all are linked by it. Interestingly, a hypoinflammatory response in serum was recently associated with chronic back pain.^56^ Here, we replicate that finding. Centrally, our results suggest an upregulation of cytokines involved in microglia activation and migration as well as, speculatively, BBB alteration. The latter is supported by the positive correlation between CSF expression of CCL11, CCL25, and CXCL9 and Alb_Q,_ a measure of BBB permeability.

#### 4.3.1. The blood–brain barrier

The BBB remains relatively elusive in the setting of pain conditions. Recently, a correlation between BBB dysfunction and expression of NFL, IL6, and IL8 in the CSF was found in patients with painful neuropathy, as well as a serum–CSF correlation and a correlation between IL6 levels and pain scores.^22^ In the present work, all but one subject has normal BBB permeability, yet the positive correlation between Alb_Q_ and NPX_Q_ and between Alb_Q_ and NPX_CSF_ indicate that even subtle, subclinical increases in BBB permeability may be of importance. Furthermore, males had significantly higher Alb_Q_ than females, but the clinical relevance of this finding remains elusive.

### 4.4. Inflammatory proteins in the cerebrospinal fluid are associated with symptoms

Cytokines of special interest are those that are significantly upregulated in CSF of patients compared with controls, whose expression correlate between serum and CSF, and whose expression correlate with the patients' clinical symptoms. CCL11, CD5, IL8, and MMP-10 meet these criteria in the LDH group, suggesting disease-specific mechanisms (Fig. 2). Surprisingly, cytokine expression levels correlate not with the neuropathic component, but with the nociceptive one (Table 8). Furthermore, instead of a linear correlation, as documented in OA patients,^53^ the associations look u-shaped (Fig. 1). These patterns must be interpreted cautiously as the subpopulation with severe back pain (group 3, Fig. 1) is small, reflecting the recruitment process.

Previous studies reporting aberrant CSF concentrations of these cytokines in LDH (IL8), OA (CCL11, CD5, IL8, and MMP-10), neuropathic pain (CCL11 and IL8), and fibromyalgia (CCL11 and IL8) establish their importance in chronic pain.^6–9,11,34,40,52,53^ In addition, CCL11 and IL8 concentrations were inversely associated with symptom severity in a cohort of patients with OA,^38,53^ suggesting modulatory effects on neuroimmunity that are not exclusively exacerbating. IL8 acts on neurons, glia, and the BBB,^43,60^ and has been implicated in reduction of neuronal output^60^ and BBB dysfunction.^10,12,27^ CCL11 has documented effects on glia and neurons,^30,54,58,72^ and has been implicated in multiple sclerosis.^54^ Its effects in chronic pain conditions and on the BBB remain elusive.

CD5 is implicated in neuroimmune disorders,^15,48^ but its CNS actions are unknown to our knowledge. MMP-10 is upregulated by TGFb^29^ and expressed by at least neurons, astrocytes, and activated microglia in the CNS,^21^ and implicated in Alzheimer's disease.^17,21^ Although we could not replicate this, Liu et al.^44^ recently reported a negative correlation between CSF-levels of TGF-b and pain in osteoarthritis, indicating a possible role for this signaling pathway in the neuroimmune modulation at least in some nociceptive pain conditions.

To summarize, the cytokines that are significantly upregulated compared with controls, correlate between serum and CSF levels, and correlate with back pain are only found in the LDH group, which suggests disease-specific mechanisms. Their common mechanisms include BBB disruption (CCL11^72^ and IL8^10,12,27^) and microglia activation (CCL11^30,54,58^ and IL-8^43,60^).

## 5. Conclusions

Elevated levels of several cytokines were found in the CSF of patients with LDH and patients with DDD, indicating neuroimmune activation, while systemic expression of inflammatory proteins suggest a hypoinflammatory environment. Furthermore, neuroimmune signaling across the BBB was suggested by correlations of cytokine levels between CSF and serum in both groups, as well as an association between cytokine levels in the CSF and albumin quotients. Complex associations were found between cytokines in CSF and back pain intensity in the LDH group, indicating disease- and context-dependent effects, with possible analgesic elements.

## 6. Limitations

This study suffers from several limitations. First, being cross-sectional, no conclusions regarding causality can be drawn. Second, for ethical reasons, we were not allowed to obtain CSF from healthy subjects and thus had to rely on 2 separate control groups. This weakness is exacerbated by not having BMI numbers for the CSF controls. Third, only surgical patients were included. Because they likely present with more severe symptoms, the question of generalization to nonsurgical LDH and DDD patients remains open. Furthermore, it would have been of great value to have another group of patients with radiologically confirmed DDD but no pain. Fourth, while one advantage of drawing the blood and CSF samples on the day of surgery is that the patients had refrained from NSAIDs for 2 weeks, administered premedication could affect the results and a higher stress level in the patient group on the day of surgery might influence levels of inflammatory proteins. Furthermore, the human immune system consists of many more active substances than the ones analyzed in our panel. Finally, cytokines generally have several downstream mechanisms of action that are multifaceted and often context-dependent, making the interpretation of our results in mechanistic terms speculative.

## Conflict of interest statement

The authors have no conflict of interest to declare.

## Appendix A. Supplemental digital content

Supplemental digital content associated with this article can be found online at http://links.lww.com/PAIN/B991.

## Supplementary Material

SUPPLEMENTARY MATERIAL
