# Supplementary material for: Unraveling the neuroimmune interface in chronic pain—the association between cytokines in the cerebrospinal fluid and pain in patients with lumbar disk herniation or degenerative disk disease
Source: Pain. 2024 Feb 5;165(7):e65–79. doi: 10.1097/j.pain.0000000000003175 (PMC11190896; doi:10.1097/j.pain.0000000000003175)
Supplement: SUPPLEMENTARY MATERIAL [file jop-165-e65-s001.pdf]

Supplemental digital content

Supplemental file A – the NINS cohort

1. List of subject sex, age and corresponding complaint

List of subject sex, age, and corresponding diagnosis

**Gender**	**Age**	**Diagnos Spec**
F	44	Tension headache
F	27	Paresthesia
F	72	Migraine
F	51	Vertigo
M	54	Paresthesia
F	61	Paresthesia
M	61	Unspecific White Matter lesions
F	59	Vertigo
F	61	Vertigo
F	26	Sensory symptoms
M	31	Sensory symptoms
M	51	Paresthesia
F	48	Dizziness / Paresthesia
M	46	Dizziness
F	41	Tension headache
F	42	Paresthesia
M	57	Tension headache
F	32	Tension headache
M	29	Paresthesia
F	49	Hypesthesia
F	33	Fatigue
F	65	Paresthesia
M	70	Visual disturbance
M	47	Lesions on fibularis nerve
F	30	Balance disturbance
F	28	Vertigo
M	56	Fatigue
M	45	paresthesia
M	42	paresthesia
F	51	Overstrained / exhaustion
M	29	Dizziness
F	56	Paresthesia
M	47	Paresthesia
F	58	Paresthesia
F	28	Tinglings in arms and legs
F	73	Labyrinth dysfunction
M	36	Papilledema
M	34	Paresthesia

M	52	Paresthesia
F	72	Paresthesia

2. Summary of symptoms/diagnoses

**Symptom / reason for neurological workup**	**N**
Balance disturbance	1
Dizziness	2
Dizziness / Paresthesia	1
Fatigue	2
Hypoesthesia	1
Labyrinth dysfunction	1
Lesions on fibularis nerve	1
Migraine	1
Overstrained / exhaustion	1
Papilledema	1
Paresthesia	15
Sensory symptoms	2
Tension headache	4
Tinglings in arms and legs	1
Unspecific White Matter lesions	1
Vertigo	4
Visual disturbance	1
**Sum**	**40**

Supplemental file B – complete protein list and PEA values from the OLINK panel

LOD = Limit of detection.
LLOQ = Lowest Level of Quantification

Protein name (CSF)	Gene	UniProt No	LOD (pg/ml)	LLOQ (pg/ml)
Eukaryotic translation initiation factor 4E binding protein 1	4EBP1	Q13541	NA	NA
Adenosine deaminase	ADA	P00813	0,48	0,48
Artemin	ARTN	Q5T4W7	0,24	0,48
Axin-1	AXIN1	O15169	61	61
Brain-derived neurotrophic factor	BDNF	P23560	NOT ANALYZED	
Beta-nerve growth factor	bNGF	P01138	0,48	0,48
Caspase-8	CASP8	Q14790	0,48	0,48
Eotaxin-1	CCL11	P51671	3,8	3,8
C-C motif chemokine 19	CCL19	Q99731	15	15
C-C motif chemokine 20	CCL20	P78556	7,6	7,6
C-C motif chemokine 23	CCL23	P55773	31	31
C-C motif chemokine 25	CCL25	O15444	3,8	3,8
C-C motif chemokine 28	CCL28	Q9NRJ3	61	122
C-C motif chemokine 3	CCL3	P10147	0,06	0,06
C-C motif chemokine 4	CCL4	P13236	1,9	1,9
Natural killer cell receptor 2B4	CD244	Q9BZW8	0,06	0,06
CD-40-L receptor	CD40	P25942	0,01	0,01
T-cell surface glycoprotein CD5 isoform	CD5	P06127	0,06	0,12
T-cell surface glycoprotein CD6 isoform	CD6	Q8WWJ7	0,24	0,24
CUB domain-containing protein 1	CDCP1	Q9H5V8	0,12	0,12
Macrophage colony stimulating factor 1	CSF1	P09603	NA	0,01
Cystatin D	CST5	P28325	1,9	1,9
Fractalkine	CX3CL1	P78423	15,3	15,3
C-X-C motif chemokine 1	CXCL1	P09341	3,8	7,6
C-X-C motif chemokine 10	CXCL10	P02778	7,6	7,6
C-X-C motif chemokine 11	CXCL11	O14625	7,6	31
C-X-C motif chemokine 5	CXCL5	P42830	0,95	0,95
C-X-C motif chemokine 6	CXCL6	P80162	7,6	31
C-X-C motif chemokine 9	CXCL9	Q07325	0,95	0,95
Delta and Notch-like epidermal growth-factor related receptor	DNER	Q8NFT8	0,95	1,9
Protein S100-A12	ENRAGE	P80511	122	122
Fibroblast growth factor 19	FGF19	O95750	7,6	7,6
Fibroblast growth factor 21	FGF21	Q9NSA1	31	31
Fibroblast growth factor 23	FGF23	Q9GZV9	122	122
Fibroblast growth factor 5	FGF5	Q8NF90	1,9	1,9

Fms-related tyrosine kinase 3 ligand	Flt3L	P49771	0,01	0,01
Glial cell-line derived neurotrophic factor	GDNF	P39905	0,01	0,01
Hepatocyte growth factor	HGF	P14210	7,6	7,6
Interferon gamma	IFN-y	P01579	15,3	15,3
Interleukin 10	IL10	P22301	0,48	0,48
Interleukin 10 receptor subunit alpha	IL10RA	Q13651	3,8	7,6
Interleukin 10 receptor subunit beta	IL10RB	Q08334	0,12	0,12
Interleukin 12 subunit beta	IL12B	P29460	0,12	0,12
Interleukin 13	IL13	P35225	7,6	7,6
Interleukin 15 receptor subunit alpha	IL15RA	Q13261	0,95	0,95
Interleukin 17 A	IL17A	Q16552	3,8	7,6
Interleukin 17 C	IL17C	Q9P0M4	31	31
Interleukin 18	IL18	Q14116	0,06	0,06
Interleukin 18 receptor 1	IL18R1	Q13478	0,06	0,06
Interleukin 1 alpha	IL1a	P01579	0,48	0,95
Interleukin 2	IL2	P60568	30,5	30,5
Interleukin 20	IL20	Q9NYY1	7,6	15
Interleukin 20 receptor subunit alpha	IL20RA	Q9UHF4	1,9	1,9
Interleukin 22 receptor subunit alpha-1	IL22RA1	Q8N6P7	0,24	0,24
Interleukin 24	IL24	Q13007	1,9	3,8
Interleukin 2 receptor subunit beta	IL2RB	P14784	15	31
Interleukin 33	IL33	O95760	3,8	3,8
Interleukin 4	IL4	P05112	0,24	0,24
Interleukin 5	IL5	P05113	3,8	3,8
Interleukin 6	IL6	P05231	0,12	0,12
Interleukin 7	IL7	P13232	0,24	0,24
Interleukin 8	IL8	P10145	0,03	0,03
Latency-associated peptide transforming growth factor beta-1	LAPTGFb	P01137	61	61
Leukemia inhibitory factor	LIF	P15018	3,8	7,6
Leukemia inhibitory factor receptor	LIFr	P42702	30,5	15,3
Monocyte chemotactic protein 1	MCP1	P13500	0,03	0,03
Monocyte chemotactic protein 2	MCP2	P80075	0,06	0,06
Monocyte chemotactic protein 3	MCP3	P80098	0,48	0,48
Monocyte chemotactic protein 4	MCP4	Q99616	7,6	7,6
Matrix metalloprotease 1	MMP1	P03956	1,9	3,9
Matrix metalloprotease 10	MMP10	P09238	0,95	0,95
Neurturin	NRTN	Q99748	3,8	7,6
Neurotrophin 3	NT3	P20783	0,12	0,12
Osteoprotegerin	OPG	O00300	0,24	0,48
Oncostatin-M	OSM	P13725	0,03	0,03
Programmed cell death 1 ligand 1	PDL1	Q9NZQ7	3,8	3,8
Stem cell factor	SCF	P21583	1,9	3,8

SIR-2-like protein 2	SIRT2	Q8IXJ6	7,6	15,3
Signaling lymphocytic activation molecule	SLAMF1	Q13291	31	31
Sulfotransferase 1A1	ST1A1	P50225	244	244
STAM-binding protein	STAMPB	O95630	7,6	7,6
Transforming growth factor alpha	TGFa	P01135	0,48	0,48
Tumor necrosis factor	TNF	P01375	0,48	0,48
Tumor necrosis factor beta	TNFB	P01374	0,24	0,48
Tumor necrosis factor receptor superfamily member 9	TNFRSF9	Q07011	0,03	0,03
Tumor necrosis factor ligand superfamily member 14	TNFSF14	O43557	0,95	1,9
TNF-related apoptosis-inducing ligand	TRAIL	P50591	0,95	0,95
TNF-related activation-induced cytokine	TRANCE	O14788	3,8	3,8
Thymic stromal lymphopoietin	TSLP	Q969D9	3,8	3,8
Tumor necrosis factor (ligand) superfamily, member 12	TWEAK	Q4ACW9	1,9	1,9
Urokinase-type plasminogen activator	uPA	P00749	0,12	0,12
Vascular endothelial growth factor A	VEGFA	P15692	0,06	0,06

Supplemental file C – CSF analyses

Protein levels

Gene	DDD (n = 40)				LDH (n = 39)				NINS (n = 37)			
	Min	Max	Mean	SD	Min	Max	Mean	SD	Min	Max	Mean	SD
4EBP1	1,03	3,42	1,705	0,426	0,69	2,49	1,689	0,382	0,64	2,34	1,345	0,430
ADA	2,30	4,30	3,338	0,443	1,27	4,81	3,214	0,581	1,27	4,64	3,003	0,618
ARTN	BELOW LIMIT OF DETECTION											
AXIN1	BELOW LIMIT OF DETECTION											
BDNF	NOT DETECTABLE DUE TO TECHNICAL ISSUES											
bNGF	1,04	2,21	1,687	0,277	0,96	2,40	1,680	0,295	0,86	2,33	1,455	0,345
CASP8	BELOW LIMIT OF DETECTION											
CCL11	1,05	2,37	1,603	0,314	1,05	2,22	1,659	0,279	1,05	2,21	1,489	0,309
CCL19	6,99	9,99	8,448	0,736	6,98	9,71	8,560	0,727	5,01	9,77	7,530	1,025
CCL20	BELOW LIMIT OF DETECTION											
CCL23	1,51	2,73	2,204	0,319	0,93	3,16	2,248	0,419	0,80	2,84	1,900	0,430
CCL25	0,62	1,23	0,798	0,187	0,62	1,61	0,862	0,272	0,62	1,69	0,788	0,268
CCL28	0,96	2,00	1,140	0,219	0,96	1,84	1,207	0,235	0,96	1,77	1,139	0,183
CCL3	1,77	3,30	2,497	0,374	1,52	3,90	2,537	0,433	1,24	4,22	2,449	0,588
CCL4	2,54	5,18	3,497	0,522	2,42	4,90	3,446	0,508	1,48	4,33	3,294	0,564
CD244	0,00	1,14	0,518	0,305	0,00	1,50	0,611	0,425	0,00	1,49	0,523	0,398
CD40	6,15	8,08	7,498	0,388	6,62	8,47	7,384	0,383	6,09	8,45	7,327	0,506
CD5	0,42	1,56	0,813	0,279	0,28	2,75	0,830	0,422	0,28	1,33	0,638	0,318
CD6	BELOW LIMIT OF DETECTION											
CDCP1	2,78	4,28	3,504	0,371	2,56	4,26	3,422	0,482	2,33	4,89	3,541	0,525
CSF1	6,06	7,59	7,009	0,319	6,14	7,68	6,966	0,335	5,72	7,84	6,862	0,453
CST5	4,78	5,30	5,107	0,103	4,97	5,29	5,139	0,070	4,83	5,24	5,028	0,107
CX3CL1	1,70	3,15	2,359	0,344	1,53	3,08	2,305	0,382	1,13	3,01	1,977	0,453
CXCL1	3,55	5,13	4,210	0,416	2,62	6,22	4,427	0,520	2,37	4,86	4,091	0,548
CXCL10	6,45	9,04	7,686	0,701	5,93	11,10	7,963	0,956	3,97	9,30	7,008	1,081
CXCL11	1,64	4,12	2,680	0,591	1,41	5,81	2,840	0,848	0,90	4,26	2,312	0,784
CXCL5	2,92	4,73	3,919	0,405	3,17	4,66	3,825	0,364	2,44	4,79	3,705	0,463
CXCL6	1,69	4,33	3,179	0,568	1,59	4,67	3,490	0,586	1,02	3,87	2,733	0,683
CXCL9	1,24	5,20	2,825	0,825	1,29	5,95	2,845	0,896	1,16	5,50	2,799	1,044
DNER	9,15	10,15	9,669	0,173	9,30	9,93	9,634	0,141	9,12	9,89	9,580	0,181
ENRAGE	BELOW LIMIT OF DETECTION											
FGF19	3,61	5,30	4,424	0,471	3,34	5,51	4,462	0,457	2,24	5,28	3,884	0,650
FGF21	BELOW LIMIT OF DETECTION											
FGF23	BELOW LIMIT OF DETECTION											
FGF5	2,55	3,86	3,294	0,331	2,45	4,03	3,193	0,403	2,09	3,95	2,999	0,421
Flt3L	7,01	8,55	7,887	0,376	6,86	8,95	7,794	0,436	6,72	8,75	7,779	0,482

GDNF	BELOW LIMIT OF DETECTION											
HGF	6,88	8,23	7,588	0,350	6,68	8,89	7,568	0,460	6,10	8,62	7,387	0,562
IFN-y	BELOW LIMIT OF DETECTION											
IL10	BELOW LIMIT OF DETECTION											
IL10RA	BELOW LIMIT OF DETECTION											
IL10RB	1,83	3,31	2,740	0,294	2,13	3,51	2,725	0,329	1,52	3,61	2,597	0,484
IL12B	0,57	1,55	0,894	0,251	0,50	3,46	0,931	0,470	0,49	2,96	0,888	0,426
IL13	BELOW LIMIT OF DETECTION											
IL15RA	BELOW LIMIT OF DETECTION											
IL17A	BELOW LIMIT OF DETECTION											
IL17C	BELOW LIMIT OF DETECTION											
IL18	0,41	1,98	1,252	0,360	0,37	2,49	1,233	0,439	0,37	2,21	1,162	0,423
IL18R1	0,52	3,01	1,802	0,569	0,96	2,99	1,803	0,408	0,74	2,67	1,620	0,504
IL1a	BELOW LIMIT OF DETECTION											
IL2	BELOW LIMIT OF DETECTION											
IL20	BELOW LIMIT OF DETECTION											
IL20RA	BELOW LIMIT OF DETECTION											
IL22RA1	BELOW LIMIT OF DETECTION											
IL24	BELOW LIMIT OF DETECTION											
IL2RB	BELOW LIMIT OF DETECTION											
IL33	BELOW LIMIT OF DETECTION											
IL4	BELOW LIMIT OF DETECTION											
IL5	BELOW LIMIT OF DETECTION											
IL6	2,23	4,79	3,377	0,663	1,86	5,46	3,342	0,666	1,82	4,52	3,066	0,582
IL7	1,01	2,03	1,280	0,227	1,01	1,77	1,248	0,186	1,01	2,08	1,270	0,269
IL8	7,14	8,73	7,753	0,391	7,28	9,05	8,010	0,448	6,48	8,73	7,616	0,511
LAPTGFb	2,67	4,64	3,828	0,394	3,29	6,61	4,084	0,566	2,55	5,32	3,997	0,742
LIF	BELOW LIMIT OF DETECTION											
LIFr	2,01	3,33	2,774	0,313	2,06	3,34	2,712	0,313	1,63	3,43	2,527	0,417
MCP1	9,80	11,70	10,801	0,447	10,11	11,57	10,895	0,388	9,56	11,74	10,782	0,468
MCP2	2,28	5,06	3,934	0,484	2,74	5,34	4,002	0,545	1,61	4,75	3,585	0,713
MCP3	BELOW LIMIT OF DETECTION											
MCP4	BELOW LIMIT OF DETECTION											
MMP1	3,28	5,75	4,700	0,560	3,26	7,20	4,871	0,813	2,58	5,80	4,525	0,795
MMP10	0,31	1,58	0,957	0,297	0,31	2,93	1,262	0,563	0,31	1,91	0,846	0,390
NRTN	BELOW LIMIT OF DETECTION											
NT3	BELOW LIMIT OF DETECTION											
OPG	9,18	10,61	9,846	0,359	9,07	10,54	9,833	0,360	8,45	10,88	9,652	0,609
OSM	BELOW LIMIT OF DETECTION											

PDL1	2,24	4,38	3,422	0,492	2,31	4,42	3,254	0,455	1,63	4,80	3,194	0,648
SCF	3,90	5,62	4,750	0,406	3,53	5,73	4,690	0,416	3,42	5,74	4,557	0,506
SIRT2	0,21	1,79	0,650	0,403	0,21	1,60	0,492	0,358	0,21	1,68	0,534	0,394
SLAMF1	BELOW LIMIT OF DETECTION											
ST1A1	BELOW LIMIT OF DETECTION											
STAMPB	BELOW LIMIT OF DETECTION											
TGFa	4,61	6,44	5,545	0,367	4,78	6,13	5,528	0,358	4,50	6,33	5,321	0,396
TNF	BELOW LIMIT OF DETECTION											
TNFB	0,16	1,08	0,525	0,281	0,16	2,09	0,487	0,379	0,16	1,47	0,427	0,306
TNFRSF9	1,32	2,33	1,847	0,284	1,28	2,89	1,883	0,380	1,02	3,00	1,846	0,419
TNFSF14	0,72	1,93	1,332	0,293	0,72	2,40	1,265	0,357	0,72	1,53	0,885	0,225
TRAIL	1,31	2,95	1,985	0,288	1,13	2,53	1,913	0,265	0,88	2,42	1,787	0,347
TRANCE	BELOW LIMIT OF DETECTION											
TSLP	BELOW LIMIT OF DETECTION											
TWEAK	9,04	11,06	10,070	0,429	9,05	11,03	9,945	0,426	8,85	11,04	9,844	0,532
uPA	6,22	8,01	7,268	0,366	6,30	8,11	7,200	0,354	5,52	8,12	6,972	0,524
VEGFA	7,73	9,82	8,895	0,434	8,18	9,66	8,829	0,432	7,61	9,71	8,558	0,487

Distribution analyses

Gene	LDH VS NINS			DDD vs NINS			LDH vs DDD		
	coef	p-value	q-value	coef	p-value	q-value	coef	p-value	q-value
4EBP1	0,44	**1,54E-06**	**1,19E-05**	0,41	**2,89E-06**	**3,27E-05**	-3,16E-02	0,71	0,90
ADA	0,25	0,06	**0,08**	0,37	4,17E-03	**0,01**	0,11	0,37	0,90
ARTN	BELOW LIMIT OF DETECTION								
AXIN1	BELOW LIMIT OF DETECTION								
BDNF	NOT DETECTABLE DUE TO TECHNICAL ISSUES								
bNGF	0,29	**3,35E-05**	**1,51E-04**	0,26	**9,16E-05**	**7,06E-04**	-2,95E-02	0,65	0,90
CASP8	BELOW LIMIT OF DETECTION								
CCL11	0,18	0,02	**0,03**	0,14	0,05	**0,08**	-3,99E-02	0,58	0,90
CCL19	1,03	**1,13E-07**	**1,52E-06**	0,98	**1,28E-07**	**3,45E-06**	-4,92E-02	0,78	0,90
CCL20	BELOW LIMIT OF DETECTION								
CCL23	0,39	**1,62E-05**	**8,77E-05**	0,33	**1,11E-04**	**7,49E-04**	-5,90E-02	0,49	0,90
CCL25	0,15	0,02	**0,03**	0,05	0,40	0,48	-9,77E-02	0,10	0,69
CCL28	0,06	0,30	0,33	-3,73E-03	0,94	0,96	-6,05E-02	0,26	0,90
CCL3	0,18	0,08	0,11	0,10	0,31	0,39	-7,96E-02	0,43	0,90
CCL4	0,29	0,02	**0,03**	0,25	0,04	**0,06**	-4,25E-02	0,72	0,90
CD244	0,12	0,18	0,21	0,06	0,49	0,55	-6,11E-02	0,47	0,90
CD40	0,17	0,07	**0,09**	0,22	0,01	**0,02**	0,06	0,53	0,90
CD5	0,25	2,04E-03	**5,80E-03**	0,20	0,01	**0,02**	-5,53E-02	0,48	0,90
CD6	BELOW LIMIT OF DETECTION								
CDCP1	7,32E-03	0,94	0,94	4,32E-03	0,96	0,96	-3,00E-03	0,98	0,99

CSF1	0,20	0,01	**0,03**	0,19	0,01	**0,02**	-6,22E-03	0,93	0,99
CST5	0,13	**1,51E-08**	**4,08E-07**	0,09	**1,92E-05**	**1,73E-04**	-3,87E-02	0,06	0,53
CX3CL1	0,42	**3,90E-06**	**2,63E-05**	0,42	**1,45E-06**	**2,61E-05**	1,20E-03	0,99	0,99
CXCL1	0,41	**3,98E-04**	**1,27E-03**	0,16	0,14	0,20	-2,52E-01	0,02	0,24
CXCL10	1,09	**6,35E-07**	**5,71E-06**	0,76	**1,90E-04**	**8,74E-04**	-3,30E-01	0,10	0,69
CXCL11	0,67	**2,24E-04**	**8,65E-04**	0,44	9,52E-03	**0,02**	-2,25E-01	0,18	0,90
CXCL5	0,20	0,04	**0,06**	0,24	8,16E-03	**0,02**	0,04	0,62	0,90
CXCL6	0,86	**6,33E-09**	**3,42E-07**	0,50	**1,94E-04**	**8,74E-04**	-3,58E-01	7,92E-03	0,12
CXCL9	0,23	0,22	0,25	0,15	0,41	0,49	-8,43E-02	0,64	0,90
DNER	0,09	0,02	**0,03**	0,11	2,33E-03	**7,40E-03**	0,02	0,60	0,90
ENRAGE	BELOW LIMIT OF DETECTION								
FGF19	0,67	**3,24E-07**	**3,50E-06**	0,58	**3,03E-06**	**3,27E-05**	-9,01E-02	0,45	0,90
FGF21	BELOW LIMIT OF DETECTION								
FGF23	BELOW LIMIT OF DETECTION								
FGF5	0,26	5,35E-03	**0,01**	0,32	**4,12E-04**	**1,59E-03**	0,06	0,51	0,90
Flt3L	0,14	0,10	0,12	0,17	0,04	**0,07**	0,03	0,76	0,90
GDNF	BELOW LIMIT OF DETECTION								
HGF	0,30	2,49E-03	**6,57E-03**	0,25	7,15E-03	**0,02**	-4,60E-02	0,62	0,90
IFN-y	BELOW LIMIT OF DETECTION								
IL10	BELOW LIMIT OF DETECTION								
IL10RA	BELOW LIMIT OF DETECTION								
IL10RB	0,21	8,82E-03	**0,02**	0,19	0,02	**0,03**	-2,48E-02	0,75	0,90
IL12B	0,14	0,18	0,21	0,09	0,37	0,45	-5,17E-02	0,62	0,90
IL13	BELOW LIMIT OF DETECTION								
IL15RA	BELOW LIMIT OF DETECTION								
IL17A	BELOW LIMIT OF DETECTION								
IL17C	BELOW LIMIT OF DETECTION								
IL18	0,08	0,39	0,41	0,12	0,18	0,23	0,04	0,66	0,90
IL18R1	0,26	0,02	**0,03**	0,23	0,03	**0,06**	-3,31E-02	0,76	0,90
IL1a	BELOW LIMIT OF DETECTION								
IL2	BELOW LIMIT OF DETECTION								
IL20	BELOW LIMIT OF DETECTION								
IL20RA	BELOW LIMIT OF DETECTION								
IL22RA1	BELOW LIMIT OF DETECTION								
IL24	BELOW LIMIT OF DETECTION								
IL2RB	BELOW LIMIT OF DETECTION								
IL33	BELOW LIMIT OF DETECTION								
IL4	BELOW LIMIT OF DETECTION								
IL5	BELOW LIMIT OF DETECTION								
IL6	0,25	0,11	0,14	0,31	0,04	**0,06**	0,07	0,66	0,90
IL7	-8,12E-03	0,89	0,90	0,02	0,67	0,70	0,03	0,57	0,90
IL8	0,44	**3,33E-05**	**1,51E-04**	0,18	0,07	0,10	-2,65E-01	8,58E-03	0,12
LAPTGFb	0,24	0,04	**0,06**	-8,94E-02	0,43	0,49	-3,31E-01	4,45E-03	0,12
LIF	BELOW LIMIT OF DETECTION								

LIFr	0,28	**4,00E-04**	**1,27E-03**	0,28	**1,62E-04**	**8,74E-04**	6,46E-03	0,93	0,99
MCP1	0,16	0,08	0,11	0,06	0,53	0,59	-1,08E-01	0,24	0,90
MCP2	0,52	**1,22E-04**	**5,07E-04**	0,40	1,67E-03	**5,63E-03**	-1,18E-01	0,35	0,90
MCP3	BELOW LIMIT OF DETECTION								
MCP4	BELOW LIMIT OF DETECTION								
MMP1	0,38	0,03	**0,05**	0,21	0,22	0,28	-1,70E-01	0,32	0,90
MMP10	0,46	**6,60E-06**	**3,96E-05**	0,15	0,10	0,14	-3,06E-01	1,44E-03	**0,08**
NRTN	BELOW LIMIT OF DETECTION								
NT3	BELOW LIMIT OF DETECTION								
OPG	0,27	3,19E-03	**7,84E-03**	0,26	3,28E-03	**9,33E-03**	-1,24E-02	0,89	0,99
OSM	BELOW LIMIT OF DETECTION								
PDL1	0,20	0,08	0,11	0,29	9,32E-03	**0,02**	0,09	0,43	0,90
SCF	0,22	0,01	**0,03**	0,25	4,88E-03	**0,01**	0,02	0,78	0,90
SIRT2	0,10	0,27	0,29	0,19	0,03	**0,06**	0,08	0,34	0,90
SLAMF1	BELOW LIMIT OF DETECTION								
ST1A1	BELOW LIMIT OF DETECTION								
STAMP B	BELOW LIMIT OF DETECTION								
TGFa	0,26	2,56E-03	**6,57E-03**	0,25	2,47E-03	**7,41E-03**	-1,05E-02	0,90	0,99
TNF	BELOW LIMIT OF DETECTION								
TNFB	0,11	0,17	0,21	0,14	0,07	0,11	0,03	0,72	0,90
TNFRSF 9	0,07	0,40	0,42	0,04	0,64	0,68	-3,20E-02	0,68	0,90
TNFSF1 4	0,45	**2,94E-08**	**5,29E-07**	0,50	**4,24E-10**	**2,29E-08**	0,04	0,54	0,90
TRAIL	0,15	0,03	**0,04**	0,22	**7,05E-04**	**2,54E-03**	0,07	0,27	0,90
TRANC E	BELOW LIMIT OF DETECTION								
TSLP	BELOW LIMIT OF DETECTION								
TWEAK	0,23	0,02	**0,03**	0,28	3,79E-03	**0,01**	0,05	0,62	0,90
uPA	0,31	1,03E-03	**3,08E-03**	0,33	**2,28E-04**	**9,48E-04**	0,03	0,78	0,90
VEGFA	0,37	**3,77E-04**	**1,27E-03**	0,38	**1,87E-04**	**8,74E-04**	3,16E-03	0,97	0,99

Supplemental file D – serum analyses

Protein levels

Gene	DDD (n = 38)				LDH (n = 39)				HC (n = 38)			
	Min	Max	Mean	SD	Min	Max	Mean	SD	Min	Max	Mean	SD
4EBP1	4,46	10,97	7,096	1,249	5,88	10,25	7,189	0,896	5,16	9,20	6,742	0,672
ADA	2,78	4,77	3,574	0,416	2,59	4,48	3,706	0,437	3,19	4,56	3,742	0,381
ARTN	BELOW LEVEL OF DETECTION											
AXIN1	1,03	4,63	1,962	0,630	1,16	3,74	1,954	0,548	1,16	3,05	1,862	0,445
BDNF	NOT DETECTABLE DUE TO TECHNICAL ISSUES											
bNGF	1,08	2,23	1,489	0,243	1,19	1,96	1,522	0,174	1,35	3,67	1,814	0,449
CASP8	0,99	3,37	1,629	0,494	1,06	3,34	1,721	0,456	1,17	2,81	1,669	0,382
CCL11	6,35	8,80	7,689	0,565	6,91	8,69	7,846	0,364	7,55	9,42	8,301	0,444
CCL19	7,00	9,97	8,640	0,683	7,65	11,75	8,887	0,718	7,70	10,46	8,840	0,600
CCL20	2,92	5,43	3,881	0,617	3,23	8,32	4,520	0,899	2,99	6,61	4,689	0,810
CCL23	8,42	10,51	9,608	0,404	8,64	10,45	9,638	0,467	8,52	10,88	9,737	0,432
CCL25	4,52	7,06	5,855	0,588	5,37	7,19	6,208	0,499	5,22	8,55	6,489	0,652
CCL28	0,19	1,97	0,985	0,380	0,38	3,30	1,060	0,544	0,58	2,06	1,219	0,364
CCL3	4,09	6,36	4,931	0,514	3,91	6,38	5,077	0,565	4,08	6,23	5,181	0,502
CCL4	6,05	9,23	7,022	0,631	5,60	8,08	7,086	0,522	6,24	8,60	7,283	0,534
CD244	4,44	6,28	5,448	0,372	4,81	6,41	5,564	0,306	5,21	6,55	5,806	0,289
CD40	8,65	10,26	9,396	0,410	8,89	10,05	9,473	0,306	9,28	10,83	9,808	0,349
CD5	3,68	5,05	4,389	0,319	3,71	5,11	4,497	0,355	4,09	5,57	4,714	0,315
CD6	2,88	5,17	4,352	0,503	3,59	5,29	4,411	0,386	3,39	6,43	4,634	0,555
CDCP1	1,18	3,35	2,327	0,543	1,81	3,81	2,624	0,438	1,90	4,45	2,936	0,557
CSF1	6,53	8,14	7,698	0,292	7,08	8,19	7,756	0,202	7,56	8,36	7,942	0,194
CST5	4,63	8,12	5,566	0,632	4,89	7,15	5,780	0,478	5,25	7,23	6,069	0,475
CX3CL1	4,18	6,12	5,311	0,420	4,61	6,18	5,435	0,378	4,88	6,51	5,556	0,367
CXCL1	7,01	9,07	8,114	0,534	6,34	10,96	8,201	0,706	7,76	9,54	8,589	0,417
CXCL10	6,17	10,47	7,475	0,823	6,10	8,86	7,515	0,675	6,59	11,54	8,034	0,850
CXCL11	5,65	9,03	7,173	0,779	5,18	9,57	7,348	0,804	6,28	10,98	7,936	0,961
CXCL5	9,84	13,63	11,733	0,844	8,01	13,24	11,586	0,900	10,13	13,25	11,974	0,696
CXCL6	6,24	9,50	8,121	0,739	6,60	9,91	8,339	0,625	7,41	9,47	8,609	0,476
CXCL9	5,60	7,98	6,737	0,624	5,42	10,45	6,747	0,814	6,08	11,27	7,376	1,102
DNER	7,24	8,61	7,975	0,272	7,55	8,58	8,054	0,227	7,63	8,89	8,260	0,309
ENRAGE	1,88	6,24	4,047	1,037	2,26	5,98	4,196	0,920	3,19	6,46	4,466	0,766
FGF19	6,06	9,90	7,512	0,940	5,93	9,97	7,872	0,973	6,87	10,71	8,509	0,915
FGF21	2,04	8,23	4,803	1,450	2,85	9,09	5,141	1,231	2,57	7,10	4,574	1,040
FGF23	0,01	2,44	0,794	0,433	0,20	1,67	0,771	0,319	0,35	2,43	1,027	0,354
FGF5	0,84	1,20	0,939	0,115	0,84	4,09	1,042	0,513	0,84	1,51	1,142	0,198
Flt3L	6,79	9,82	8,336	0,575	7,55	9,26	8,388	0,411	8,30	10,00	9,069	0,428
GDNF	0,26	2,55	1,044	0,457	0,36	1,62	1,149	0,302	0,61	2,16	1,317	0,383
HGF	7,19	9,12	8,260	0,432	7,37	9,28	8,288	0,441	8,01	9,48	8,653	0,378
IFN-y	BELOW LEVEL OF DETECTION											
IL10	0,75	3,18	2,207	0,526	1,51	4,38	2,434	0,478	1,71	6,01	2,732	0,728

IL10RA	0,81	5,04	1,444	1,022	0,81	5,66	1,350	1,045	0,81	3,55	1,189	0,546
IL10RB	4,64	6,69	6,168	0,365	5,30	7,05	6,299	0,389	5,63	7,00	6,419	0,274
IL12B	2,57	5,70	3,910	0,673	2,07	5,73	3,964	0,810	3,15	5,32	4,251	0,553
IL13	BELOW LEVEL OF DETECTION											
IL15RA	0,86	1,50	1,001	0,132	0,86	1,46	1,038	0,131	0,86	1,79	1,100	0,169
IL17A	0,00	0,84	0,142	0,265	0,00	2,71	0,212	0,509	0,00	2,67	0,468	0,625
IL17C	0,23	1,29	0,583	0,324	0,23	2,45	0,797	0,541	0,23	2,25	1,084	0,548
IL18	6,26	8,95	7,524	0,598	6,54	8,86	7,565	0,576	5,08	9,04	7,593	0,665
IL18R1	5,86	7,82	6,767	0,470	6,11	7,64	6,903	0,419	6,07	7,92	6,867	0,338
IL1a	BELOW LEVEL OF DETECTION											
IL2	BELOW LEVEL OF DETECTION											
IL20	BELOW LEVEL OF DETECTION											
IL20RA	BELOW LEVEL OF DETECTION											
IL22RA1	BELOW LEVEL OF DETECTION											
IL24	BELOW LEVEL OF DETECTION											
IL2RB	BELOW LEVEL OF DETECTION											
IL33	BELOW LEVEL OF DETECTION											
IL4	BELOW LEVEL OF DETECTION											
IL5	BELOW LEVEL OF DETECTION											
IL6	1,11	6,41	2,490	0,938	1,43	4,06	2,583	0,629	1,46	4,76	2,965	0,765
IL7	3,13	6,00	4,552	0,560	3,41	5,77	4,639	0,503	4,04	6,45	4,993	0,525
IL8	4,14	7,90	6,054	0,646	5,13	7,20	6,150	0,512	5,82	7,79	6,646	0,486
LAPTGFb	6,54	8,54	7,634	0,472	7,16	8,40	7,811	0,320	7,53	9,38	8,177	0,469
LIF	BELOW LEVEL OF DETECTION											
LIFr	1,42	2,97	2,532	0,312	1,95	3,07	2,540	0,251	2,50	3,60	2,871	0,233
MCP1	9,22	11,87	10,434	0,611	9,62	11,61	10,464	0,427	9,98	12,31	10,781	0,474
MCP2	6,17	9,18	8,349	0,650	6,35	9,90	8,458	0,668	6,48	10,02	8,654	0,745
MCP3	0,60	8,97	1,593	1,321	0,58	2,76	1,504	0,450	0,96	4,50	1,775	0,608
MCP4	2,59	5,36	4,189	0,694	3,24	5,21	4,248	0,451	3,57	5,59	4,645	0,419
MMP1	11,71	14,87	13,495	0,883	11,88	15,11	13,639	0,872	11,63	14,95	13,911	0,856
MMP10	3,93	6,80	5,375	0,566	4,30	8,20	5,779	0,810	4,77	7,32	6,016	0,597
NRTN	BELOW LEVEL OF DETECTION											
NT3	0,29	2,43	0,858	0,407	0,31	2,03	0,958	0,404	0,41	3,14	1,316	0,550
OPG	8,57	11,09	9,877	0,478	8,94	10,60	9,962	0,375	9,76	11,05	10,299	0,288
OSM	2,21	5,49	3,492	0,741	1,16	5,29	3,468	0,861	2,38	5,74	4,101	0,725
PDL1	2,34	4,43	3,331	0,391	2,86	5,90	3,596	0,527	2,97	4,70	3,615	0,337
SCF	8,75	10,21	9,485	0,365	8,80	10,17	9,606	0,347	9,30	10,43	9,909	0,284
SIRT2	0,09	4,88	1,688	1,050	0,49	3,82	1,675	0,619	0,69	2,82	1,551	0,556
SLAMF1	0,00	2,11	1,319	0,511	0,37	2,24	1,325	0,436	0,84	2,76	1,573	0,452
ST1A1	1,03	4,20	2,503	0,803	0,07	4,89	2,618	1,021	0,78	4,34	2,666	0,813
STAMPB	2,76	6,17	3,653	0,672	2,59	5,32	3,704	0,471	2,94	4,60	3,612	0,373
TGFa	2,67	5,43	3,890	0,624	2,70	4,81	3,797	0,539	3,31	5,49	4,441	0,575
TNF	BELOW LEVEL OF DETECTION											
TNFB	2,29	4,69	3,735	0,427	2,29	4,36	3,692	0,398	2,87	4,41	3,891	0,351
TNFRSF9	4,55	6,60	5,743	0,454	4,99	6,69	5,853	0,386	5,27	7,11	5,915	0,374

TNFSF14	4,49	7,04	5,768	0,577	4,31	7,07	5,707	0,623	5,25	7,22	6,076	0,501
TRAIL	7,43	9,24	8,191	0,374	7,37	9,09	8,293	0,328	7,79	9,69	8,470	0,333
TRANCE	2,80	5,58	4,149	0,701	2,73	5,69	4,237	0,602	3,45	5,97	4,383	0,590
TSLP	BELOW LEVEL OF DETECTION											
TWEAK	8,37	10,11	9,396	0,381	7,71	10,09	9,387	0,442	8,66	10,83	9,739	0,379
uPA	8,83	10,21	9,619	0,354	9,10	10,28	9,766	0,267	9,48	10,74	10,094	0,306
VEGFA	8,78	11,17	9,847	0,610	9,02	10,93	9,931	0,495	9,31	11,60	10,269	0,571

Distribution analyses

Gene	LDH vs HC			DDD vs HC			LDH vs DDD		
	**coef**	**p-value**	**q-value**	**coef**	**p-value**	**q-value**	**coef**	**p-value**	**q-value**
4EBP1	0,31	0,22	0,27	0,31	0,18	0,21	3,37E-03	0,99	0,99
ADA	-2,49E-01	0,01	**0,03**	-2,64E-01	5,21E-03	**8,68E-03**	-1,46E-02	0,87	0,97
ARTN	BELOW LEVEL OF DETECTION								
AXIN1	0,06	0,67	0,72	0,09	0,50	0,53	0,03	0,83	0,95
BDNF	NOT DETECTABLE DUE TO TECHNICAL ISSUES								
bNGF	-2,63E-01	1,39E-03	**4,74E-03**	-2,95E-01	**1,24E-04**	**3,87E-04**	-3,17E-02	0,67	0,93
CASP8	-4,42E-02	0,63	0,68	-8,97E-02	0,28	0,32	-4,55E-02	0,58	0,93
CCL11	-4,33E-01	**3,35E-04**	**1,20E-03**	-5,65E-01	**8,08E-07**	**8,66E-06**	-1,32E-01	0,22	0,93
CCL19	5,61E-04	1,00	1,00	-2,50E-01	0,14	0,17	-2,51E-01	0,13	0,91
CCL20	-4,75E-01	8,71E-03	**0,02**	-9,69E-01	**4,30E-08**	**1,07E-06**	-4,94E-01	2,93E-03	0,22
CCL23	-1,10E-01	0,34	0,39	-1,20E-01	0,26	0,30	-9,85E-03	0,93	0,98
CCL25	-1,55E-01	0,30	0,35	-5,35E-01	**1,62E-04**	**4,66E-04**	-3,79E-01	5,91E-03	0,22
CCL28	9,98E-03	0,92	0,94	-1,36E-01	0,17	0,20	-1,46E-01	0,13	0,91
CCL3	-1,57E-01	0,26	0,31	-2,54E-01	0,05	**0,06**	-9,74E-02	0,44	0,93
CCL4	-2,16E-01	0,15	0,19	-2,55E-01	0,07	**0,08**	-3,94E-02	0,77	0,93
CD244	-3,54E-01	**6,78E-05**	**4,56E-04**	-4,18E-01	**6,08E-07**	**7,60E-06**	-6,42E-02	0,41	0,93
CD40	-3,77E-01	**6,26E-05**	**4,56E-04**	-4,16E-01	**2,31E-06**	**1,45E-05**	-3,99E-02	0,63	0,93
CD5	-3,50E-01	**6,60E-05**	**4,56E-04**	-3,96E-01	**1,52E-06**	**1,04E-05**	-4,61E-02	0,55	0,93
CD6	-2,52E-01	0,05	**0,08**	-3,11E-01	8,32E-03	**0,01**	-5,85E-02	0,61	0,93
CDCP1	-2,67E-01	0,03	**0,05**	-5,39E-01	**6,02E-06**	**3,01E-05**	-2,72E-01	0,02	0,41
CSF1	-2,53E-01	**4,24E-05**	**3,98E-04**	-2,80E-01	**1,36E-06**	**1,02E-05**	-2,70E-02	0,62	0,93
CST5	-2,49E-01	0,07	0,11	-4,53E-01	**5,42E-04**	**1,27E-03**	-2,04E-01	0,11	0,89
CX3CL1	-1,93E-01	0,06	**0,09**	-2,93E-01	2,23E-03	**4,29E-03**	-1,01E-01	0,28	0,93
CXCL1	-5,67E-01	**1,28E-04**	**6,77E-04**	-5,85E-01	**2,12E-05**	**8,83E-05**	-1,85E-02	0,89	0,97
CXCL10	-4,79E-01	0,02	**0,04**	-5,20E-01	5,89E-03	**9,60E-03**	-4,11E-02	0,82	0,95
CXCL11	-6,67E-01	3,37E-03	**9,36E-03**	-8,30E-01	**9,95E-05**	**3,24E-04**	-1,63E-01	0,42	0,93
CXCL5	-4,58E-01	0,03	**0,05**	-2,85E-01	0,13	0,16	0,17	0,35	0,93
CXCL6	-4,99E-01	2,21E-03	**6,83E-03**	-6,32E-01	**3,70E-05**	**1,46E-04**	-1,33E-01	0,36	0,93
CXCL9	-4,37E-01	0,05	**0,08**	-5,05E-01	0,01	**0,02**	-6,74E-02	0,74	0,93
DNER	-2,66E-01	**1,56E-04**	**7,32E-04**	-3,07E-01	**3,34E-06**	**1,79E-05**	-4,12E-02	0,51	0,93
ENRAGE	-5,73E-01	0,01	**0,03**	-5,82E-01	6,49E-03	**0,01**	-8,62E-03	0,97	0,99
FGF19	-5,18E-01	0,04	**0,06**	-9,20E-01	**8,04E-05**	**2,74E-04**	-4,02E-01	0,07	0,89
FGF21	0,50	0,12	0,16	0,30	0,31	0,34	-2,02E-01	0,48	0,93
FGF23	-2,28E-01	0,03	**0,05**	-2,94E-01	2,02E-03	**3,99E-03**	-6,57E-02	0,48	0,93
FGF5	-6,21E-02	0,46	0,52	-1,74E-01	0,03	**0,04**	-1,12E-01	0,15	0,91

Flt3L	-6,46E-01	**3,65E-07**	**2,73E-05**	-7,25E-01	**1,66E-09**	**6,24E-08**	-7,91E-02	0,47	0,93
GDNF	-2,07E-01	0,04	**0,06**	-3,04E-01	1,13E-03	**2,29E-03**	-9,66E-02	0,28	0,93
HGF	-4,93E-01	**6,30E-06**	**1,58E-04**	-4,50E-01	**7,84E-06**	**3,46E-05**	0,04	0,65	0,93
IFN-y	BELOW LEVEL OF DETECTION								
IL10	-3,09E-01	0,05	**0,08**	-5,52E-01	**2,11E-04**	**5,47E-04**	-2,43E-01	0,09	0,89
IL10RA	0,06	0,81	0,86	0,22	0,32	0,34	0,16	0,45	0,93
IL10RB	-2,76E-01	2,17E-03	**6,83E-03**	-3,18E-01	**1,53E-04**	**4,60E-04**	-4,22E-02	0,60	0,93
IL12B	-5,32E-01	2,74E-03	**7,92E-03**	-4,90E-01	2,82E-03	**5,28E-03**	0,04	0,79	0,94
IL13	BELOW LEVEL OF DETECTION								
IL15RA	-7,24E-02	0,14	0,19	-1,15E-01	0,01	**0,02**	-4,26E-02	0,35	0,93
IL17A	-4,75E-01	**3,00E-04**	**1,12E-03**	-4,40E-01	**2,89E-04**	**7,00E-04**	0,04	0,76	0,93
IL17C	-4,69E-01	**2,05E-04**	**8,53E-04**	-5,82E-01	**1,08E-06**	**9,04E-06**	-1,13E-01	0,31	0,93
IL18	-2,69E-01	0,07	0,11	-1,63E-01	0,24	0,27	0,11	0,43	0,93
IL18R1	-1,47E-01	0,15	0,19	-1,87E-01	0,05	**0,06**	-3,95E-02	0,67	0,93
IL1a	BELOW LEVEL OF DETECTION								
IL2	BELOW LEVEL OF DETECTION								
IL20	BELOW LEVEL OF DETECTION								
IL20RA	BELOW LEVEL OF DETECTION								
IL22RA1	BELOW LEVEL OF DETECTION								
IL24	BELOW LEVEL OF DETECTION								
IL2RB	BELOW LEVEL OF DETECTION								
IL33	BELOW LEVEL OF DETECTION								
IL4	BELOW LEVEL OF DETECTION								
IL5	BELOW LEVEL OF DETECTION								
IL6	-4,16E-01	0,03	**0,05**	-4,90E-01	4,65E-03	**7,93E-03**	-7,39E-02	0,66	0,93
IL7	-3,70E-01	9,87E-03	**0,02**	-4,40E-01	9,89E-04	**2,12E-03**	-7,02E-02	0,59	0,93
IL8	-6,35E-01	**1,29E-05**	**1,93E-04**	-6,33E-01	**2,89E-06**	**1,66E-05**	2,12E-03	0,99	0,99
LAPTGF b	-4,57E-01	**7,29E-05**	**4,56E-04**	-5,61E-01	**2,67E-07**	**5,01E-06**	-1,05E-01	0,30	0,93
LIF	BELOW LEVEL OF DETECTION								
LIFr	-3,16E-01	**1,75E-05**	**2,19E-04**	-3,37E-01	**9,71E-07**	**9,04E-06**	-2,11E-02	0,74	0,93
MCP1	-3,74E-01	5,19E-03	**0,01**	-3,59E-01	3,72E-03	**6,66E-03**	0,02	0,90	0,97
MCP2	-1,96E-01	0,29	0,34	-2,45E-01	0,15	0,18	-4,91E-02	0,77	0,93
MCP3	-3,89E-01	0,08	0,12	-2,10E-01	0,31	0,34	0,18	0,38	0,93
MCP4	-4,06E-01	4,05E-03	**0,01**	-4,52E-01	**5,90E-04**	**1,34E-03**	-4,57E-02	0,72	0,93
MMP1	-3,56E-01	0,12	0,16	-4,27E-01	0,04	**0,06**	-7,05E-02	0,73	0,93
MMP10	-2,30E-01	0,16	0,20	-5,77E-01	**2,22E-04**	**5,54E-04**	-3,47E-01	0,02	0,41
NRTN	BELOW LEVEL OF DETECTION								
NT3	-4,50E-01	**1,99E-04**	**8,53E-04**	-5,11E-01	**6,50E-06**	**3,04E-05**	-6,14E-02	0,57	0,93
OPG	-2,75E-01	6,46E-03	**0,02**	-3,78E-01	**7,12E-05**	**2,54E-04**	-1,03E-01	0,26	0,93
OSM	-8,54E-01	**4,13E-05**	**3,98E-04**	-7,16E-01	**1,78E-04**	**4,78E-04**	0,14	0,45	0,93
PDL1	-7,38E-02	0,52	0,58	-3,01E-01	4,64E-03	**7,93E-03**	-2,28E-01	0,03	0,44
SCF	-3,48E-01	**1,35E-04**	**6,77E-04**	-4,39E-01	**3,85E-07**	**5,77E-06**	-9,10E-02	0,26	0,93
SIRT2	0,12	0,55	0,61	0,14	0,45	0,47	0,02	0,91	0,97
SLAMF1	-3,78E-01	2,28E-03	**6,83E-03**	-3,11E-01	6,17E-03	**9,85E-03**	0,07	0,55	0,93
ST1A1	0,02	0,92	0,94	-9,79E-02	0,64	0,65	-1,22E-01	0,56	0,93
STAMP B	0,02	0,87	0,90	0,02	0,90	0,90	-6,66E-03	0,96	0,99

TGFa	-7,01E-01	**9,21E-06**	**1,73E-04**	-5,77E-01	**6,70E-05**	**2,51E-04**	0,12	0,36	0,93
TNF	BELOW LEVEL OF DETECTION								
TNFB	-2,55E-01	0,01	**0,03**	-2,12E-01	0,02	**0,03**	0,04	0,64	0,93
TNFRSF9	-1,75E-01	0,09	0,13	-2,09E-01	0,03	**0,04**	-3,35E-02	0,72	0,93
TNFSF14	-5,21E-01	**2,55E-04**	**1,01E-03**	-3,77E-01	3,73E-03	**6,66E-03**	0,14	0,25	0,93
TRAIL	-2,14E-01	0,02	**0,04**	-2,80E-01	1,06E-03	**2,20E-03**	-6,66E-02	0,42	0,93
TRANCE	-4,07E-01	0,01	**0,02**	-3,52E-01	0,02	**0,02**	0,06	0,70	0,93
TSLP	BELOW LEVEL OF DETECTION								
TWEAK	-4,17E-01	**1,33E-04**	**6,77E-04**	-3,77E-01	**1,79E-04**	**4,78E-04**	0,04	0,68	0,93
uPA	-3,89E-01	**5,88E-06**	**1,58E-04**	-5,14E-01	**5,45E-10**	**4,09E-08**	-1,25E-01	0,10	0,89
VEGFA	-4,28E-01	3,86E-03	**0,01**	-4,57E-01	9,04E-04	**1,99E-03**	-2,86E-02	0,83	0,95

Supplemental file E – sex differences in protein expression

Estimate = median NPX difference (female – male). A positive estimate indicates higher levels in females, while a negative estimate indicates higher levels in males.

Serum

Protein	DDD			LDH		
	Estimate	p-value	q-value	Estimate	p-value	q-value
4E-BP1	-0,69	0,078	0,506	0,38	0,938	0,988
ADA	-0,23	0,024	0,506	-0,11	0,631	0,988
AXIN1	0,08	0,601	0,932	-0,08	0,816	0,988
Beta-NGF	0,05	0,542	0,924	0,04	0,230	0,867
CASP-8	-0,07	0,179	0,610	-0,06	0,889	0,988
CCL11	-0,08	0,862	1,000	-0,25	0,114	0,817
CCL19	0,04	0,486	0,924	-0,75	0,000	**0,018**
CCL20	-0,02	0,399	0,901	-0,40	0,653	0,988
CCL23	-0,05	0,542	0,924	-0,13	0,121	0,817
CCL25	-0,17	0,294	0,856	0,01	0,963	0,988
CCL28	0,10	0,152	0,573	0,26	0,312	0,867
CCL3	-0,22	0,908	1,000	-0,31	0,195	0,862
CCL4	-0,16	0,367	0,901	0,06	0,914	0,988
CD244	-0,11	0,161	0,573	-0,03	0,816	0,988
CD40	-0,14	0,432	0,901	0,16	0,816	0,988
CD5	-0,46	0,068	0,506	-0,07	0,609	0,988
CD6	-0,23	0,024	0,506	-0,01	0,631	0,988
CDCP1	-0,22	0,467	0,924	-0,09	0,376	0,910
CSF-1	0,19	0,161	0,573	-0,02	0,745	0,988
CST5	-0,14	0,308	0,856	-0,12	0,699	0,988
CX3CL1	0,18	0,706	0,963	0,01	0,566	0,988
CXCL1	0,44	0,045	0,506	0,10	0,393	0,921
CXCL10	-0,28	0,322	0,863	0,30	0,653	0,988
CXCL11	-0,13	1,000	1,000	0,13	0,447	0,958
CXCL5	0,41	0,772	0,993	0,00	0,816	0,988
CXCL6	0,58	0,089	0,506	-0,27	0,914	0,988
CXCL9	-0,14	0,161	0,573	0,09	0,359	0,910
DNER	-0,06	0,308	0,856	-0,14	0,174	0,817
EN-RAGE	0,17	0,977	1,000	-0,03	0,653	0,988
FGF-19	0,81	0,036	0,506	0,19	0,769	0,988
FGF-21	-0,19	0,794	0,993	0,02	0,914	0,988
FGF-23	0,14	0,862	1,000	0,23	0,164	0,817
FGF-5	-0,06	0,431	0,901	0,06	0,145	0,817
Flt3L	0,10	0,095	0,506	0,32	0,030	0,751
GDNF	0,07	0,977	1,000	0,08	0,256	0,867
HGF	0,12	0,504	0,924	-0,10	0,566	0,988

IL-10RA	0,16	0,373	0,901	-0,13	0,263	0,867
IL-10RB	-0,05	1,000	1,000	-0,03	0,889	0,988
IL-12B	-0,35	0,152	0,573	0,04	0,988	0,988
IL-15RA	0,02	0,953	1,000	-0,06	0,158	0,817
IL-17A	0,12	0,393	0,901	-0,06	0,119	0,817
IL-17C	-0,23	0,085	0,506	-0,03	0,925	0,988
IL-18R1	-0,22	0,581	0,932	-0,36	0,174	0,817
IL10	-0,12	0,337	0,871	-0,13	0,312	0,867
IL18	-0,44	0,019	0,506	-0,42	0,019	0,729
IL5	0,27	0,036	0,506	0,13	0,756	0,988
IL6	0,43	0,561	0,932	0,07	0,722	0,988
IL7	0,07	0,685	0,963	-0,06	0,769	0,988
IL8	-0,10	0,294	0,856	-0,43	0,071	0,817
LAP TGF-beta-1	-0,05	0,931	1,000	-0,20	0,327	0,877
LIF-R	-0,12	0,794	0,993	0,09	0,230	0,867
MCP-1	0,17	0,601	0,932	-0,18	0,312	0,867
MCP-2	0,03	0,885	1,000	0,06	0,676	0,988
MCP-3	-0,23	0,622	0,932	-0,05	0,963	0,988
MCP-4	0,33	0,622	0,932	0,10	0,447	0,958
MMP-1	0,20	0,885	1,000	-0,29	0,376	0,910
MMP-10	-0,03	0,706	0,963	-0,09	0,631	0,988
NT-3	-0,21	0,294	0,856	-0,11	0,914	0,988
OPG	0,01	0,523	0,924	0,20	0,174	0,817
OSM	0,22	1,000	1,000	0,39	0,988	0,988
PD-L1	-0,20	0,078	0,506	-0,02	0,889	0,988
SCF	-0,13	1,000	1,000	-0,16	0,938	0,988
SIRT2	0,21	0,908	1,000	0,14	0,699	0,988
SLAMF1	-0,01	0,750	0,993	-0,24	0,269	0,867
ST1A1	-0,21	0,794	0,993	0,61	0,297	0,867
STAMPB	0,01	0,152	0,573	0,04	0,988	0,988
TGF-alpha	-0,01	0,663	0,957	-0,06	0,963	0,988
TNFB	-0,03	0,839	1,000	-0,09	0,699	0,988
TNFRSF9	-0,35	0,064	0,506	-0,18	0,076	0,817
TNFSF14	-0,01	0,523	0,924	-0,14	0,609	0,988
TRAIL	-0,17	0,152	0,573	-0,15	0,066	0,817
TRANCE	-0,24	0,052	0,506	-0,30	0,081	0,817
TWEAK	-0,21	0,663	0,957	0,05	0,243	0,867
uPA	-0,10	0,432	0,901	0,05	0,411	0,933
VEGFA	-0,35	0,486	0,924	0,15	0,816	0,988

CSF

Protein	DDD Estimate	DDD p-value	DDD q-value	LDH Estimate	LDH p-value	LDH q-value
4E-BP1	-0,05	0,757	0,965	-0,23	0,286	0,499
ADA	-0,15	0,657	0,965	-0,13	0,590	0,797
Beta-NGF	-0,06	0,697	0,965	-0,08	0,794	0,912
CCL11	-0,08	0,095	0,853	-0,23	0,116	0,369
CCL19	-0,49	0,037	0,853	-0,65	0,014	0,251
CCL20	-0,01	0,663	0,965	-0,10	0,380	0,569
CCL23	-0,11	0,476	0,965	-0,09	0,272	0,499
CCL25	-0,16	0,486	0,965	0,02	0,839	0,917
CCL28	-0,17	0,054	0,853	-0,14	0,138	0,382
CCL3	-0,14	0,366	0,965	-0,26	0,046	0,338
CCL4	0,15	0,286	0,965	-0,09	0,866	0,917
CD244	-0,25	0,079	0,853	-0,48	0,005	0,136
CD40	0,02	0,819	0,965	0,04	0,656	0,843
CD5	0,07	0,778	0,965	-0,07	0,198	0,412
CDCP1	0,13	0,180	0,965	0,15	0,939	0,939
CSF-1	0,13	0,638	0,965	-0,18	0,198	0,412
CST5	-0,01	0,840	0,965	0,06	0,259	0,499
CX3CL1	0,11	0,757	0,965	-0,26	0,149	0,382
CXCL1	0,24	0,581	0,965	-0,10	0,198	0,412
CXCL10	0,32	0,510	0,965	-0,55	0,083	0,367
CXCL11	0,12	0,338	0,965	-0,44	0,363	0,559
CXCL5	0,04	0,737	0,965	-0,05	0,842	0,917
CXCL6	-0,16	0,968	0,968	-0,37	0,158	0,387
CXCL9	0,07	0,737	0,965	-0,42	0,089	0,367
DNER	-0,03	0,638	0,965	-0,04	0,301	0,501
FGF-19	-0,02	0,968	0,968	-0,08	0,747	0,912
FGF-5	0,01	0,925	0,968	0,22	0,272	0,499
Flt3L	0,16	0,199	0,965	-0,16	0,109	0,367
HGF	0,06	0,638	0,965	-0,01	0,590	0,797
IL-10RB	0,02	0,968	0,968	-0,17	0,149	0,382
IL-12B	0,05	0,717	0,965	0,01	0,914	0,932
IL-18R1	-0,06	0,946	0,968	-0,22	0,286	0,499
IL18	-0,42	0,009	0,491	0,04	0,315	0,501
IL6	0,15	0,819	0,965	-0,37	0,198	0,412
IL7	0,04	0,644	0,965	-0,05	0,628	0,827
IL8	0,03	0,619	0,965	-0,22	0,058	0,338
LAP TGF-beta-1	-0,03	0,325	0,965	-0,40	0,058	0,338
LIF-R	0,10	0,325	0,965	0,02	0,794	0,912
MCP-1	-0,05	0,619	0,965	-0,56	0,063	0,338
MCP-2	0,07	0,527	0,965	-0,32	0,109	0,367

MMP-1	0,05	0,396	0,965	-0,02	0,315	0,501
MMP-10	0,06	0,737	0,965	-0,28	0,040	0,338
OPG	-0,07	0,545	0,965	-0,31	0,054	0,338
PD-L1	0,18	0,352	0,965	0,10	0,866	0,917
SCF	-0,09	0,581	0,965	-0,22	0,102	0,367
SIRT2	-0,02	0,828	0,965	0,15	0,522	0,742
TGF-alpha	-0,17	0,527	0,965	0,02	0,450	0,657
TNFB	0,12	0,624	0,965	-0,27	0,126	0,378
TNFRSF9	-0,14	0,657	0,965	-0,39	0,001	**0,044**
TNFSF14	0,13	0,366	0,965	0,01	0,724	0,909
TRAIL	-0,19	0,095	0,853	-0,22	0,078	0,367
TWEAK	-0,07	0,882	0,968	0,14	0,770	0,912
uPA	0,10	0,381	0,965	-0,31	0,029	0,338
VEGFA	-0,12	0,925	0,968	-0,09	0,890	0,924
